# Data-driven nanomechanical sensing: specific information extraction from a complex system

**DOI:** 10.1038/s41598-017-03875-7

**Published:** 2017-06-16

**Authors:** Kota Shiba, Ryo Tamura, Gaku Imamura, Genki Yoshikawa

**Affiliations:** 1 World Premier International Research Center Initiative (WPI), International Center for Materials Nanoarchitectonics (MANA), National Institute for Materials Science (NIMS), 1-1 Namiki, Tsukuba, Ibaraki, 305-0044 Japan; 2 Center for Materials Research by Information Integration (CMI2), National Institute for Materials Science (NIMS), 1-2-1 Sengen, Tsukuba, Ibaraki, 305-0047 Japan; 3 International Center for Young Scientists (ICYS), National Institute for Materials Science (NIMS), 1-1 Namiki, Tsukuba, Ibaraki, 305-0044 Japan; 4 Materials Science and Engineering, Graduate School of Pure and Applied Science, University of Tsukuba, Tennodai 1-1-1 Tsukuba, Ibaraki, 305-8571 Japan

## Abstract

Smells are known to be composed of thousands of chemicals with various concentrations, and thus, the extraction of specific information from such a complex system is still challenging. Herein, we report for the first time that the nanomechanical sensing combined with machine learning realizes the specific information extraction, *e*.*g*. alcohol content quantification as a proof-of-concept, from the smells of liquors. A newly developed nanomechanical sensor platform, a Membrane-type Surface stress Sensor (MSS), was utilized. Each MSS channel was coated with functional nanoparticles, covering diverse analytes. The smells of 35 liquid samples including water, teas, liquors, and water/EtOH mixtures were measured using the functionalized MSS array. We selected characteristic features from the measured responses and kernel ridge regression was used to predict the alcohol content of the samples, resulting in successful alcohol content quantification. Moreover, the present approach provided a guideline to improve the quantification accuracy; hydrophobic coating materials worked more effectively than hydrophilic ones. On the basis of the guideline, we experimentally demonstrated that additional materials, such as hydrophobic polymers, led to much better prediction accuracy. The applicability of this data-driven nanomechanical sensing is not limited to the alcohol content quantification but to various fields including food, security, environment, and medicine.

## Introduction

Quantification is an important process in most of the analyses. Many approaches have been developed for centuries to precisely derive values that are characteristic of an analyte. Volume, weight, density, and concentration, for example, are representative ones and there are currently lots of techniques available to measure these values. A prerequisite, in most cases, is that an analytical target has to be a single component or, at least, the target can be analyzed independent of the other components. For this reason, chromatography is normally used to obtain an individual component from a complicated mixture, allowing for the quantification of each component. Thus, it has been a long standing challenge to directly analyze complicated samples and to quantitatively extract specific values without a separation process.

One of the representative examples of a complex sample is a smell; it is known that most of the smells are composed of thousands of chemicals or more. Such numerous constituents contain various information which reflects their origin, attracting people to pursue potential applications of smells in diverse fields^1–4^. The chromatographic approach is a straightforward way to the quantitative analysis of the smells; however, it is time-consuming and requires trained experts. On the other hand, various sensor arrays that consist of multiple sensors with different chemical properties have been developed as a tool for the smell analysis^5–8^ as most living things including mammals adopt a similar approach^9, 10^. Each sensor element in the sensor array responds differently when exposed to the smell because of their diverse chemical properties, resulting in a unique pattern which can be regarded as a fingerprint of the smell. This type of approach, so-called a pattern recognition, is useful for discriminating a sample from others. In addition to the discrimination, the possibilities of the quantification were investigated on the basis of the multiple sensor responses^11^, whereas it has been experimentally proved to be practically impossible to directly and quantitatively extract specific values in a complex sample with more than three components by a conventional approach.

**Figure 1:**
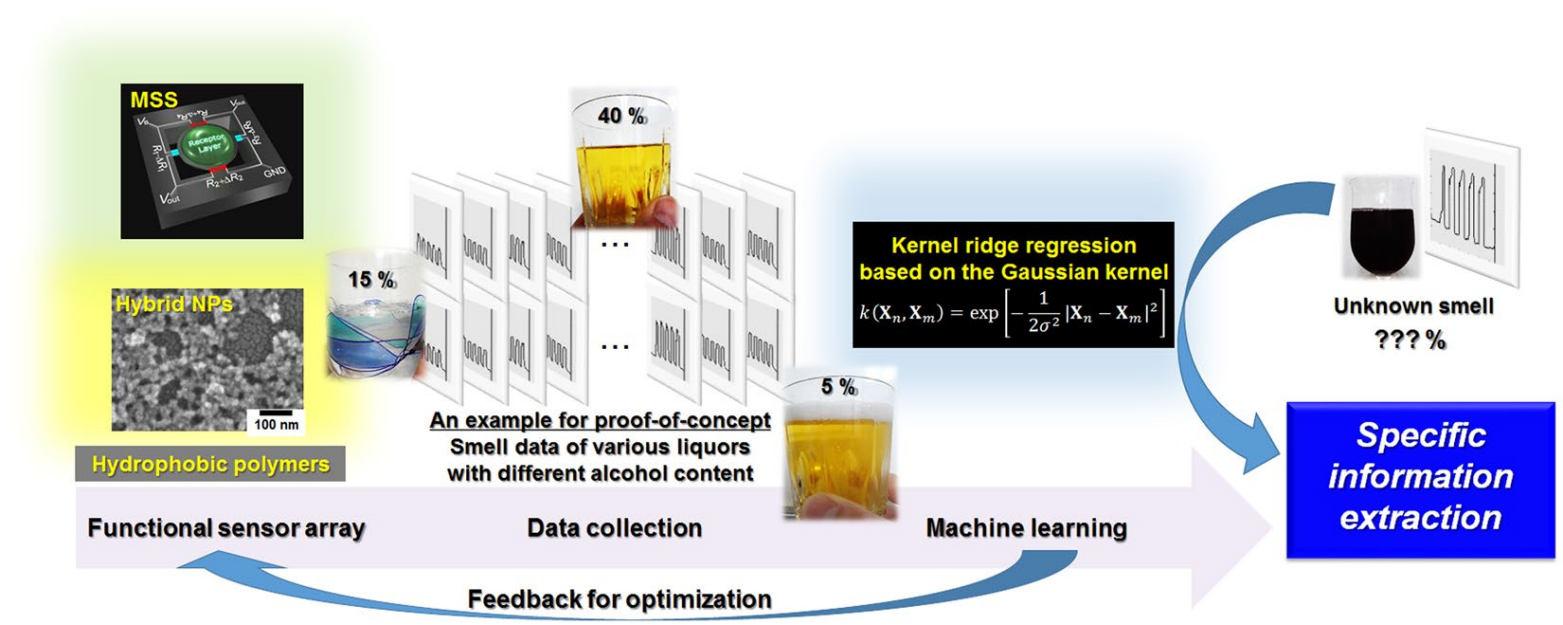
A concept of the present study. Note that the alcohol content is just an example of the specific information for a proof-of-concept.

In the present study, we demonstrate that a sensor array combined with a machine learning technique can be utilized to derive quantitative information, *e*.*g*. alcohol content, from the smells of various liquors (Fig. 1). A Membrane-type Surface stress Sensor (MSS) with four independent channels was used as a model sensing system^12^. As reported previously, MSS has several advantages over similar sensing techniques including those based on conventional cantilever-type nanomechanical sensors with optical/piezoresistive readout; high sensitivity^13^, compact system, and stable operation^14, 15^ in gas/liquid phase. It is important to note that the MSS structure is much more robust against the fluctuation of the receptor layers compared to a cantilever structure, providing a reliable dataset for advanced analysis including machine learning^15^. According to the previously reported analytical guideline to improve the sensitivity^16^, we coated each channel of MSS with receptor materials having large Young’s moduli, *i*.*e*. functional silica/titania hybrid nanoparticles, that were synthesized by means of a multi-step nucleation controlled growth method^17^. These nanoparticles with different chemical properties rendered the MSS capable of discriminating variety of chemicals, providing an advanced sensor platform to challenge the long-standing issue; the extraction of specific information (*e*.*g*. alcohol concentration in the present case) from a complex system (*e*.*g*. smells). The machine learning technique is another important key on the software side to achieving the quantitative extraction. In this study, we used kernel ridge regression, since it can be applied to a nonlinear problem with avoiding overfitting. The combination of the advanced sensor platform with the machine learning technique led to the successful extraction of specific information.

## Results and Discussion

### Preparation of silica/titania hybrid nanoparticles with different surface functionalities

**Figure 2:**
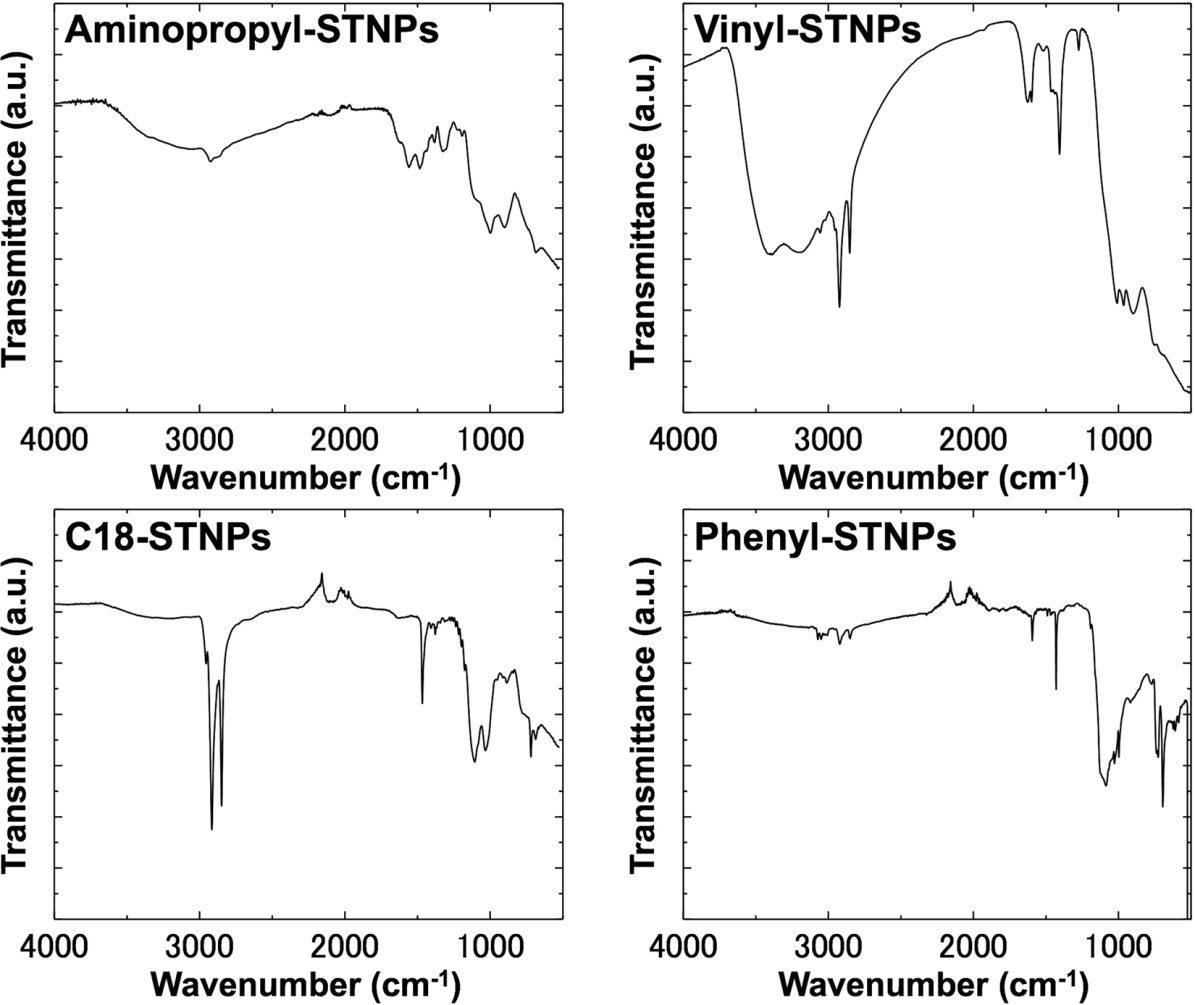
FT-IR spectra of four different types of NPs used in the present study: Aminopropyl-STNPs, Vinyl-STNPs, C18-STNPs, and Phenyl-STNPs.

**Figure 3:**
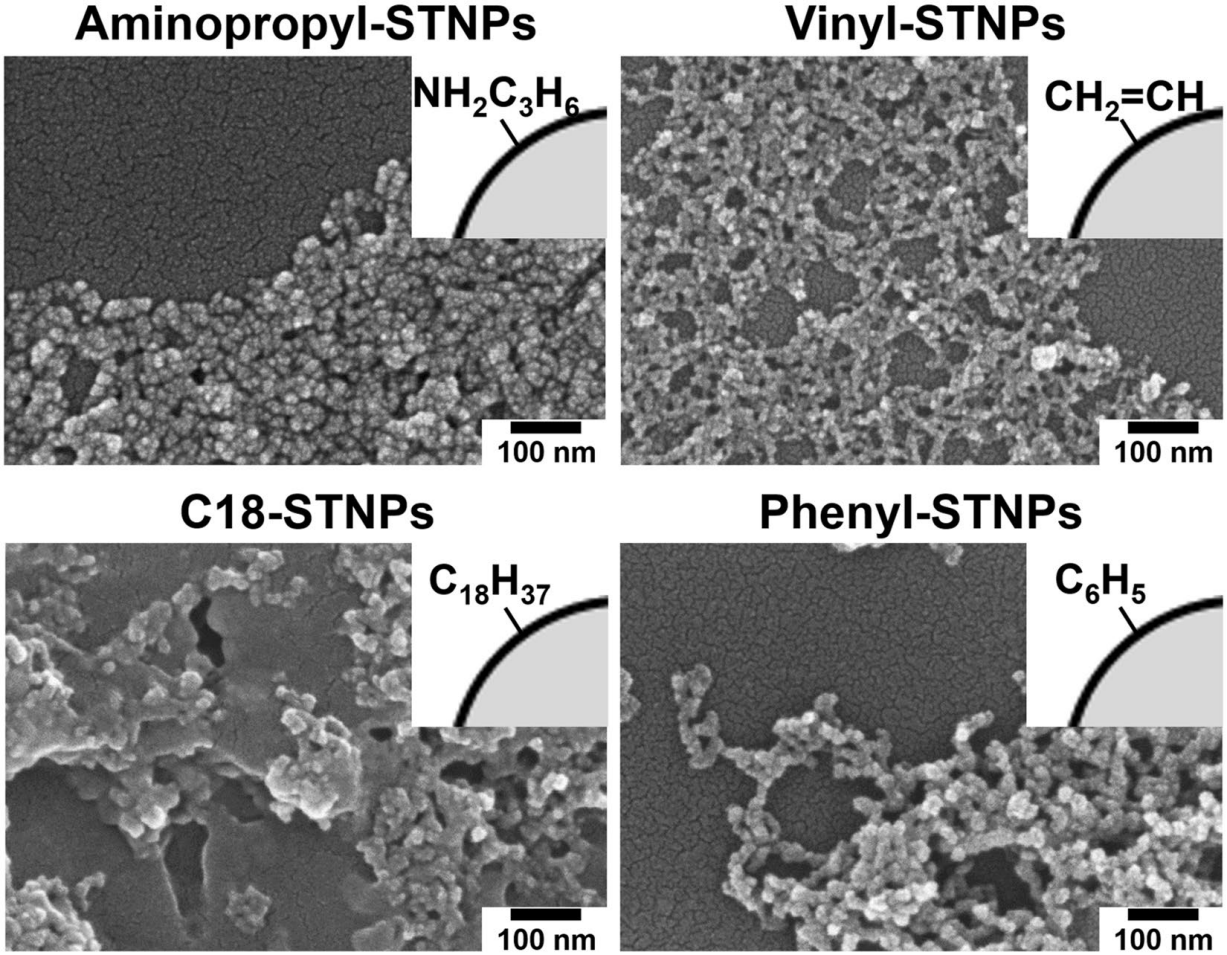
SEM images of four different types of NPs used in the present study: Aminopropyl-STNPs, Vinyl-STNPs, C18-STNPs, and Phenyl-STNPs.

Silica/titania-based hybrid nanoparticles (NPs) with different surface functionalities were prepared through the hydrolysis and co-condensation reaction of two alkoxides, *i*.*e*. titanium tetraisopropoxide (TTIP) and various silane coupling reagents, combined with a multi-step microfluidic approach which we reported previously (details are described in Methods section)^17^. In the present study, four types of functional groups (aminopropyl group, vinyl group, octadecyl group, and phenyl group) were immobilized on the product surface. To confirm the presence of these functional groups, all the samples were characterized by FT-IR spectroscopy as shown in Fig. 2. Briefly, the FT-IR spectrum of the vinyl group modified sample exhibited two characteristic absorption bands at 1406 cm^−1^ and 1600 cm^−1^; the former is attributed to the –CH_2_ in-plane bending deformation and the latter to the C=C stretching mode^18, 19^. The FT-IR spectrum of the octadecyl group modified sample showed intense absorption bands at 2848 and 2916 cm^−1^ that can be ascribed to the C–H stretching vibrations of the octadecyl groups^20^. The absorption bands appeared at 1430 cm^−1^ and 738 cm^−1^ come from the phenyl group covalently attached to Si, while the absorption bands appeared at 1594, 1571, 1490, 1067, 1027, and 694 cm^−1^ stem from various vibration modes of the phenyl group itself^21^. As for the aminopropyl group modified sample, the spectrum shown in Fig. 2 matches well with that reported previously^17^, indicating the successful aminopropyl functionalization. Almost no absorption band being characteristic of the hydrogen bonding network is recognized at around 3000–3500 cm^−1^ for the octadecyl group and phenyl group modified samples, reflecting the intrinsic hydrophobicity of these functional groups. In contrast, a clear absorption band appears at the same wavenumber region for aminopropyl group and vinyl group modified samples. According to previous studies^22, 23^, the FT-IR spectra for aminopropyl functionalized solids usually show the similar trend because of their hydrophilic surface property. However, vinyl functionalized solids are basically considered to be hydrophobic. Taking account of the amount of immobilized vinyl groups quantified by a thermogravimetric analysis, there are still a large number of accessible hydroxyl groups, probably resulting in the present hydrophilicity. SEM images shown in Fig. 3 reveal that the four samples have the average size of around a few tens of nm, thereby being denoted as “NPs” hereafter standing for nanoparticles. The corresponding size distributions seem narrow enough to conclude that there are no by-products formed by homogeneous nucleation and subsequent growth of either TTIP or silane coupling reagents, indicating that the silane-based functional groups were co-precipitated with titania to form the functional NPs. The NPs with different surface functionalities are denoted as Aminopropyl-STNPs, Vinyl-STNPs, C18-STNPs, and Phenyl-STNPs, respectively.

### Sensing properties of various NPs coated MSS under an ambient condition

To investigate the performance of each functional NP as a receptor layer material for nanomechanical sensing, the NPs were coated on the surface of MSS by a spray coating method (details are described in Methods section). The whole sensor surface was covered with the coating layer as confirmed by an optical microscope (Figure S1). The coating thickness was estimated to be approximately 1 μm. As C18-STNPs and Phenyl-STNPs did not well-disperse in the mixture of water/IPA, some aggregates are recognized in the corresponding optical microscope images.

The sensing performance of the NPs-coated MSS under an ambient condition was examined by measuring headspace gases of 15 chemical compounds that can be roughly classified into following six categories: water-based, alkane, alcohol, aromatic, ketone, and others (details are described in Methods section). As a result, each sensor responds differently to the chemicals (Figure S2). The response trend can be interpreted based on the expected property of the coated NPs. For example, Aminopropyl-STNPs and Vinyl-STNPs covered the water-based ones and alcohols, while C18-STNPs and Phenyl-STNPs covered the aromatic compounds. The C18-STNPs showed the highest affinity with the alkanes because of their structural similarity in the functional group.

**Figure 4:**
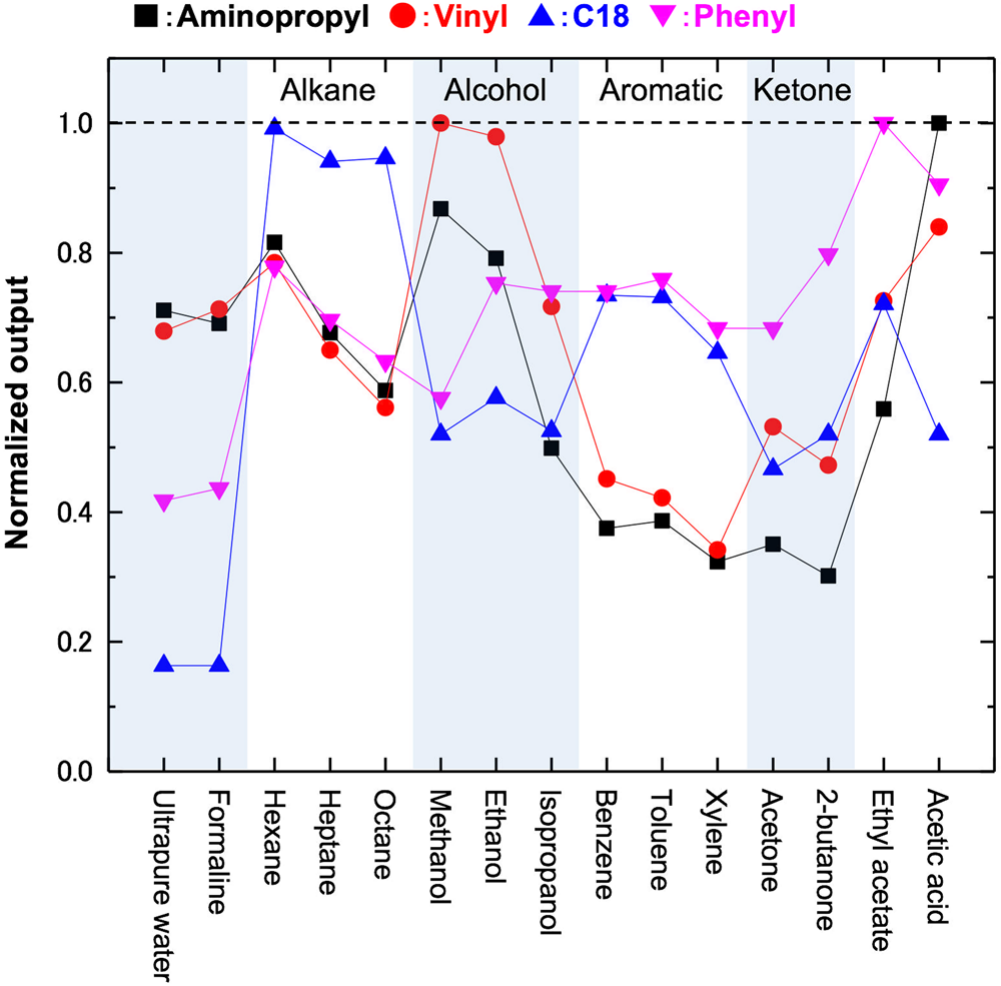
Affinity trend of four different types of NPs used in the present study: Aminopropyl-STNPs, Vinyl-STNPs, C18-STNPs, and Phenyl-STNPs. The normalized output used in this figure was defined on the basis of the signal intensity of the third response cycle shown in Figure S2 (the first and second responses were omitted to get reproducible responses because the initial instability was observed in the first two cycles).

**Figure 5:**
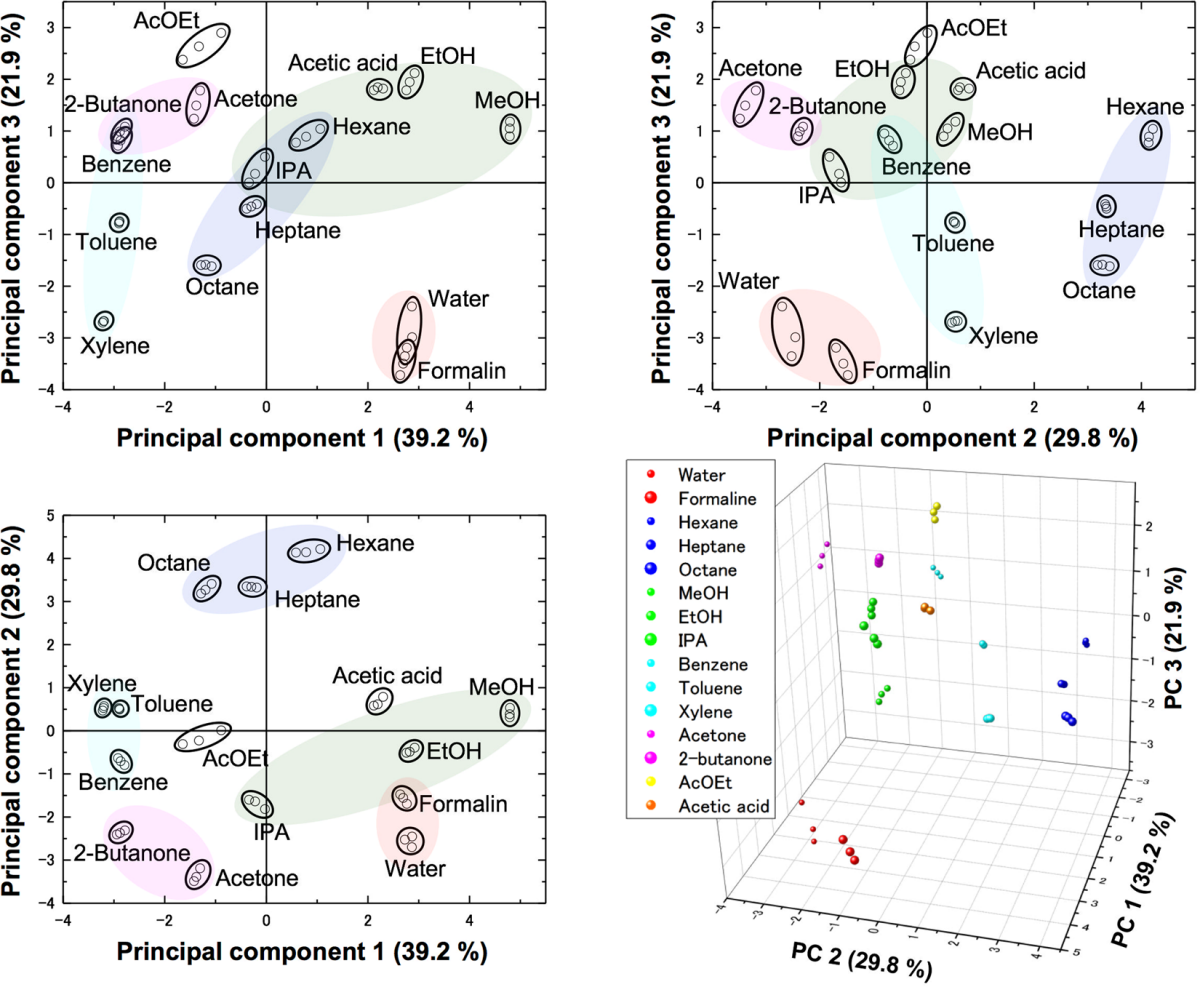
PCA plots to discriminate the 15 chemicals using the NPs-coated MSS. (Top left, top right, and bottom left) The 2D plot with the two principal components. (Bottom right) The 3D plot drawn based on the three principal components. The PCA shown here was performed utilizing four parameters extracted from the last three measured responses shown in Figure S2. The details on the extraction procedure are explained later.

As each channel was able to cover different types of chemicals complementarily (Fig. 4), the combination of the present NPs should possess a sufficient discrimination property when they are utilized as a receptor layer material for sensing purpose. To prove this assumption, we performed a principal component analysis (PCA) on the data set obtained from the measurements of the 15 chemicals. The PCA score plots shown in Fig. 5 demonstrate that the chemicals are clearly discriminated depending on their chemical structures.

### Machine learning-based prediction of alcohol content of various liquors

As demonstrated in the previous section, the MSS with differently functionalized channels achieved the discrimination of various chemicals by extracting simple parameters from the measured responses (details will be explained later). This chemical diversity makes it possible to derive quantitative information from complicated smells. In this study, we focused on the smells of various liquors as a model sample, predicting alcohol content of unknown liquors by means of machine learning.

**Figure 6:**
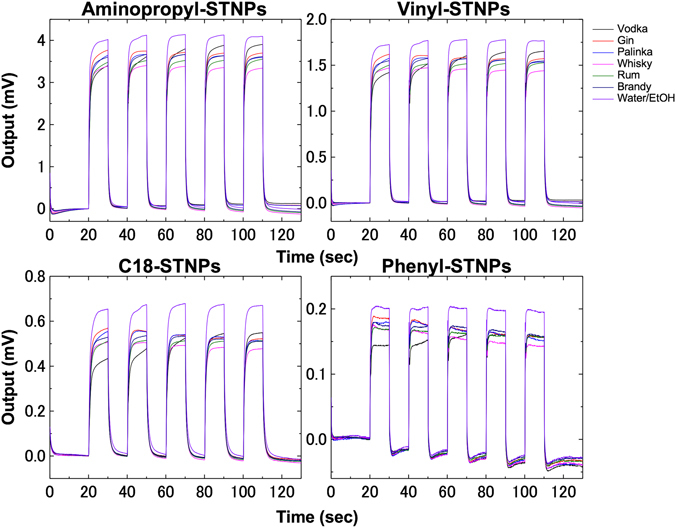
Response signals from the liquid samples with the same alcohol content (40%). The shape/intensity of the responses are different even with the same alcohol content because of the difference in other various components such as flavors.

We confirmed that the responses to measuring the smells of 35 liquid samples including commercial liquors, non-alcoholic liquids, and simple water/EtOH mixtures were different in terms of their shape and intensity (Figure S3). As shown in Fig. 6, for example, the response signals are not the same even among the liquors with the same alcohol contents. These results indicate that the extraction of alcohol content is impossible through a simple analysis of the response signals; *e*.*g*. comparing peak heights. One of the previous studies pointed out that it is practically impossible to experimentally determine the concentration of each component in the system containing more than three components^11^. Thus, in the present study, the correlation among signals was trained through the machine learning (ML) technique, focusing on the alcohol content. We constructed the ML model to predict an alcohol content of a liquor from any signal measured by the MSS.

**Figure 7:**
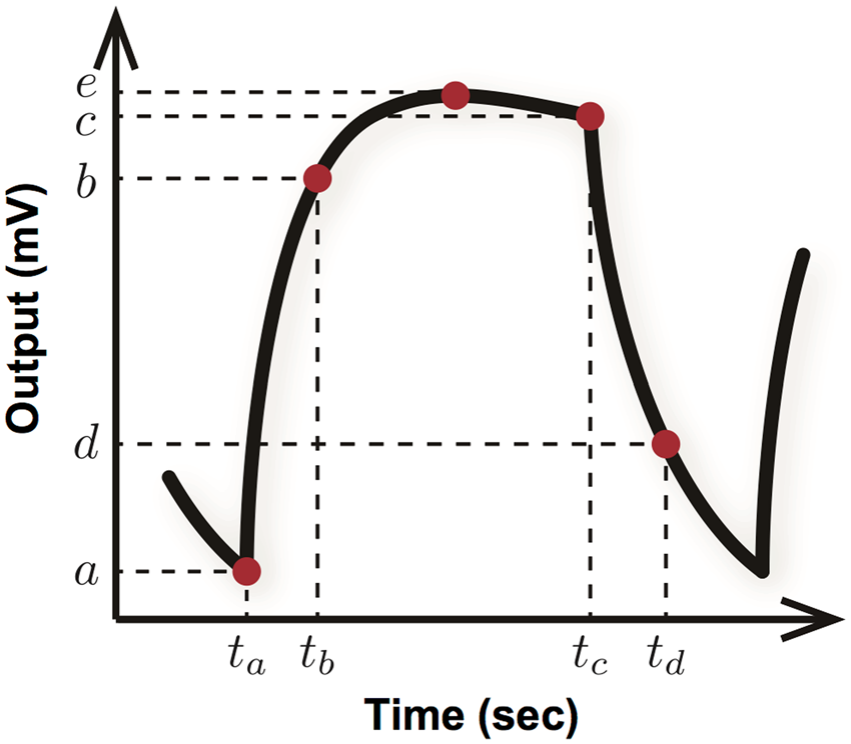
Schematic of a feature extraction of a signal measured by the MSS. Four parameters are defined as features by using $a,\,b,\,c,\,d,\,e,\,{t}_{a},\,{t}_{b},{t}_{c},$ and ${t}_{d}$.

We used kernel ridge regression (KRR)^24^ as a machine learning technique. The hyper parameters in KRR were decided such that the prediction error Δ, which is defined in Methods section, obtained by performing the cross validation is minimized. The details of KRR and cross validation are described in Methods section, respectively. To perform KRR, features of a signal measured by the MSS were expressed by four parameters which are defined as follows:

1 $${\rm{Parameter}}\,1:(b-a)/({t}_{b}-{t}_{a})$$

2 $${\rm{Parameter}}\,2:(c-b)/({t}_{c}-{t}_{b})$$

3 $${\rm{Parameter}}\,3:(d-c)/({t}_{d}-{t}_{c})$$

4 $${\rm{Parameter}}\,4:(e-a)$$

where $a,\,b,\,c,\,d,\,e,\,{t}_{a},\,{t}_{b},{t}_{c},$ and *t*_*d*_ are denoted in Fig. 7. In this case, ${t}_{b}=\,{t}_{a}+1\,$[s], ${t}_{c}=\,{t}_{a}+10\,$[s], and ${t}_{d}=\,{t}_{a}+11\,$[s] were used. Three sets of the parameters were extracted from the latter three signals where *t*_*a*_ = 60, 80, and 100 out of the five signals in the response signals, since the latter cycles could provide reproducible signals without initial fluctuations such as mixing of sample gases and pre-adsorbed gases. The parameter 1 should reflect the adsorption process of analytes, while the parameter 3 should include the information on the desorption process. The parameter 2 was defined as a slope in-between the adsorption/desorption processes, being regarded as a quasi-equilibrium state. The parameter 4 was defined as the maximum height obtained from each response signal, reflecting the adsorption capacity-related property of the coating layer.

**Figure 8:**
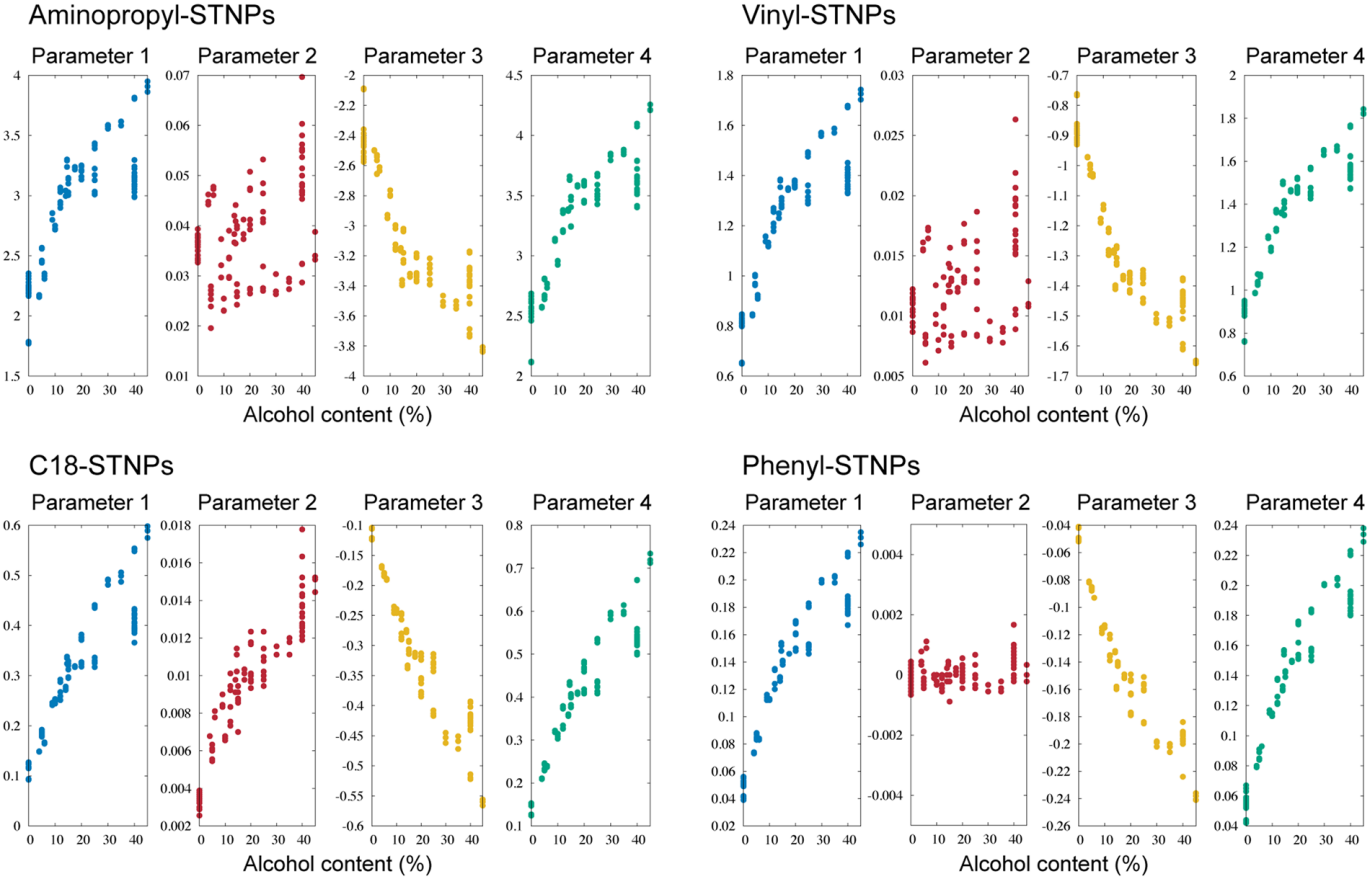
Alcohol content dependence of the parameters extracted from response signals under an ambient condition.

Figure 8 is the alcohol content dependence of the parameters extracted from the signals of the 35 liquid samples measured by the MSS under an ambient condition (details are described in Method section). In each liquid sample, three signals where *t*_*a*_ = 60, 80, and 100 were used, and thus, 105 data points exist in Fig. 8. Except the parameter 2, moderate correlations of parameters with respect to the alcohol content were confirmed. Thus, the parameters 1, 3, and 4 are useful to train the ML model for the alcohol content. On the other hand, at this stage, we were not able to conclude that which receptor layer materials were suitable for estimating the alcohol content.

**Figure 9:**
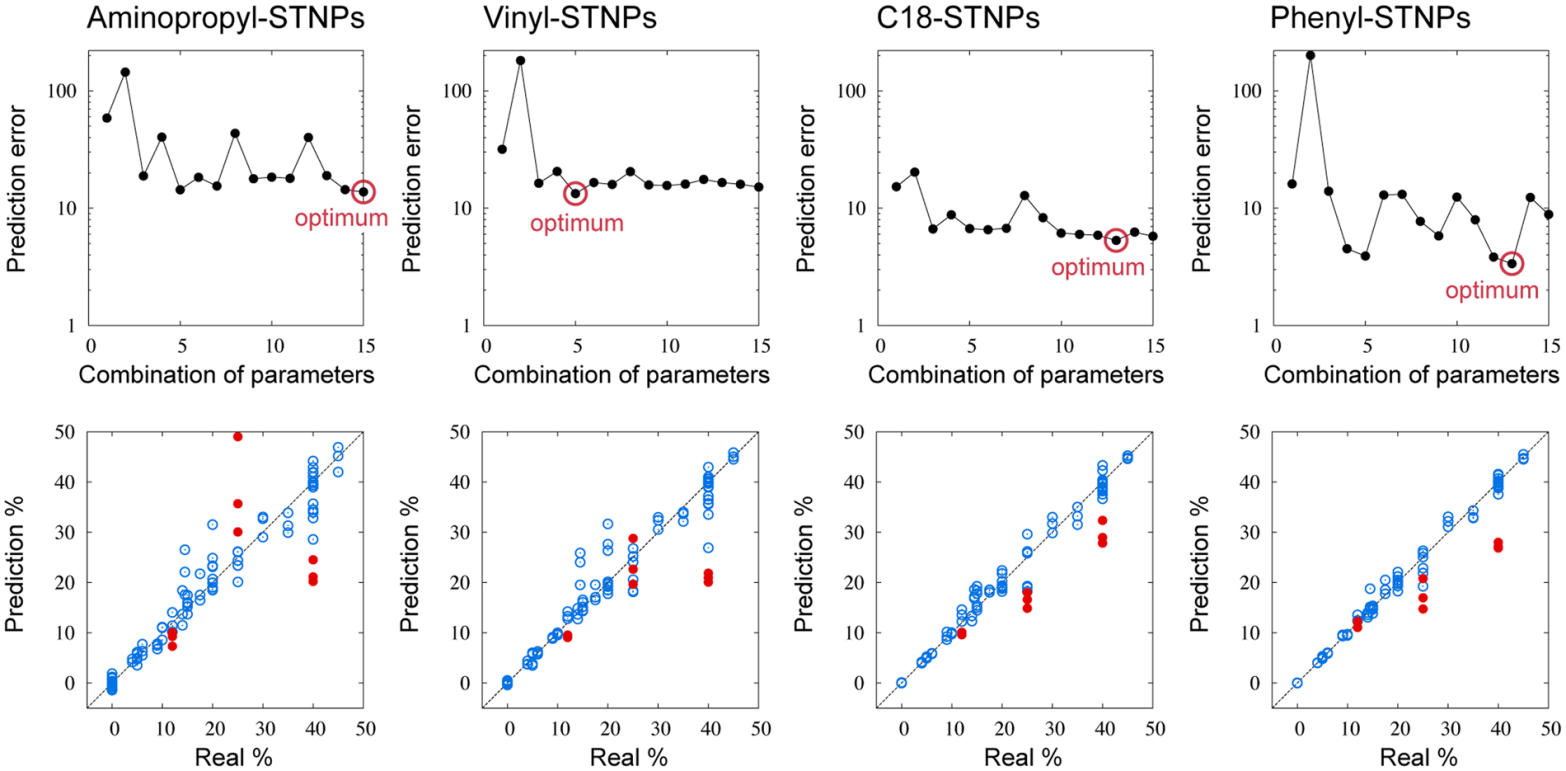
(Top) Prediction errors depending on the combination of four parameters extracted from a signal under an ambient condition. The combination of parameters is labeled by the decimal number. In the binary representation of the decimal number, the 1st digit bit corresponds to the use of parameter 1 (0: “not used”, 1: “used”); same for 2nd, 3rd, and 4th digit bits for parameters 2, 3, and 4, respectively. For example, the number of the combination of parameters 13 corresponds to 1101 in the binary representation, meaning that the parameters 1, 3, and 4 are used. (Bottom) Parity plot of predicted alcohol content versus real alcohol content under an ambient condition. The blue points represent the known liquors which are used to train the ML model. The red points are the unknown liquors: red wine (12%), imo-shochu (25%), and whisky (40%).

By using these extracted parameters, we performed KRR for the responses of each receptor layer material. We first analyzed the case that a single sensor channel in the MSS was used. We trained ML models to predict the alcohol content of liquors for all combinations of the four parameters; accordingly, the number of trained ML models for each receptor layer material was 15 = 2^4^ − 1. The 32 liquid samples out of 35 (except red wine, imo-shochu, and whisky) were used for the training as a known dataset. We trained the ML model by 96 signals where three signals were used in each liquid sample. Top figures of Fig. 9 show the prediction error Δ depending on the combination of parameters when the 24-fold cross validation was adopted. The optimal value of the prediction error in each receptor layer material and the optimal combination of parameters are summarized in Table 1. It is suggested that C18-STNPs and Phenyl-STNPs are more useful to predict the alcohol contents of liquors compared to Aminopropyl-STNPs and Vinyl-STNPs. In addition, it was confirmed that parameter 2 was not informative to predict an alcohol content. Furthermore, we show the parity plot of predicted alcohol content versus actual alcohol content in bottom figures in Fig. 9 when we used the optimal combination of the parameters. The blue points denote the known liquid samples which are used to train the ML model and the red points are the unknown liquors: red wine, imo-shochu, and whisky. For the known liquors, the prediction by the ML model was successful when a receptor layer material was C18-STNPs or Phenyl-STNPs, while Aminopropyl-STNPs and Vinyl-STNPs showed much larger prediction errors. Judging from these results, it seems important to use hydrophobic receptor layer materials to predict the alcohol content of liquors. On the other hand, the prediction performance for the unknown liquor was still not sufficient even if C18-STNPs or Phenyl-STNPs was used. It should be noted here that, by using the ML technique and evaluating the prediction error, effective receptor materials can be selected depending on the target. We also confirmed that the same results were obtained in the case under a N_2_ environment (results are described in Supplementary Figure (Figure S4) and Supplementary Note sections).

**Table 1:** Optimal combination of parameters and optimal prediction error depending on the receptor layer material under an ambient condition.

	Aminopropyl	Vinyl	C18	Phenyl
Parameter 1	Use	Use	Use	Use
Parameter 2	Use			
Parameter 3	Use	Use	Use	Use
Parameter 4	Use		Use	Use
Prediction error	13.7373	13.2925	5.3103	3.3576

**Figure 10:**
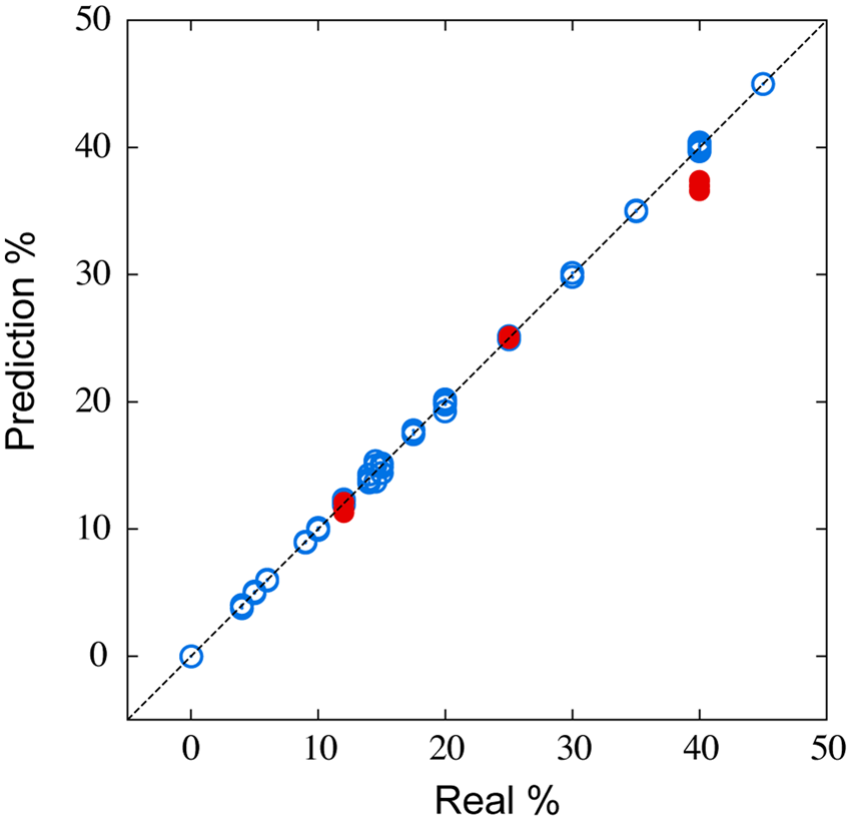
Parity plot of predicted alcohol content versus real alcohol content under an ambient condition when the parameter 3 of Phenyl-STNPs, the parameter 4 of polysulfone, and the parameters 1 and 3 of polycaprolactone were used. The blue points represent the known liquors which are used to train the ML model. The red points are the unknown liquors: red wine (12%), imo-shochu (25%), and whisky (40%).

To improve the prediction performance further, we investigated the case of training the ML model using the MSS with all four sensor channels coated with hydrophobic materials. In addition to C18-STNPs and Phenyl-STNPs, we utilized two commercial hydrophobic polymers: polysulfone and polycaprolactone (in Supplementary Figures (Figures S5, S6 and S7) and Supplementary Note sections, all the data including measured responses, and the training results by using each polymer are shown.). In this case, since the features of a signal by each polymer were also expressed by the four parameters explained above, the 16 parameters were obtained from the four sensor channels in the MSS. The ML models to predict the alcohol content of liquors were constructed for all combinations of the 16 parameters; accordingly the number of trained ML models was 65535 = 2^16^ − 1. By performing 24-fold cross validation, prediction errors Δ for all the combinations were evaluated, and the optimal combination was found. In the optimal combination (the parameter 3 of phenyl, the parameter 4 of polysulfone, and the parameters 1 and 3 of polycaprolactone), the prediction error was Δ = 0.4315, meaning that the prediction performance was drastically improved than those in the cases using a single sensor channel shown in Fig. 9 and Table 1. Figure 10 is the parity plot of predicted alcohol content versus real alcohol content when the optimal combination was used. We found that the prediction by the ML model was successful not only for the known liquors but also for the unknown liquors with high accuracy. This result clearly demonstrates the significant capability of extracting target quantity from complex systems through the combination of a multi-channel chemical sensor and machine learning.

Finally, we examined the usage rate of each parameter in top 100 combinations when four channels in the MSS were used in Table 2. The prediction errors in the top 100 combinations were distributed in between Δ = 0.4315 and 0.5735. From this table, we confirmed that the usage rate of the parameter 2 was small except for the case of polysulfone (the correlation of the parameter 2 with respect to the alcohol content was clearly indicated only in the polysulfone case, which is described in Supplementary Note). Furthermore, while the usage rate of the parameter 3 was larger than that of the parameter 1 in C18-STNPs and Phenyl-STNPs cases, the inverse result was obtained in polysulfone and polycaprolactone cases. These results suggest that the present NPs coatings derived more information from the desorption process than the adsorption process, while the polymers showed the opposite trend. To interpret such trends, we need to consider physicochemical properties of the NPs and polymers. According to the previous literature, one dominant factor to determine the shape of the responses is the ratio between sorption and diffusion time constants^25^. Since the NPs coatings should possess a number of pores that are formed by NP-NP interstices. The porous structures would allow for faster sorption than that of the polymers, making it difficult to derive much information under the present parameter extraction condition. On the other hand, the desorption behavior would be similar to that from a capillary where a hysteresis is usually observed for mesoporous materials with their typical pore size ranging from 2 nm to 50 nm^26, 27^. Thus, the retarded desorption should provide more information in the form of parameter 3. In the case of the polymers, it takes longer time for both sorption and desorption because of their denser structures than the NPs-based porous structure. Consequently, useful information for the prediction was extracted from both parameters 1 and 3. Needless to say, some chemical interactions between the receptor coatings and adsorbates also affect the present results. Thus, the feature extraction based on physical/chemical behaviors will be effective for further discussions^7^, which we are going to report elsewhere.

**Table 2:** Usage rate of each parameter in top 100 combinations when four sensor channels are used.

	C18	Phenyl	Polysulfone	Polycaprolactone
Parameter 1	17%	10%	74%	90%
Parameter 2	0%	0%	36%	2%
Parameter 3	58%	52%	63%	47%
Parameter 4	39%	47%	79%	66%

In summary, we realized the prediction of alcohol content of various liquors based on their smells that are usually composed of thousands of constituents. Taking advantage of a nanomechanical sensor platform, MSS, functionalized with four different types of NPs in combination with a machine learning technique, the alcohol content was accurately predicted from such complicated samples. The machine learning model indicated that the NPs with hydrophobic property such as octadecyl and phenyl groups-modified NPs significantly contributed to the accurate prediction. Thus, we experimentally confirmed that the use of additional two polymers with hydrophobic properties further improved the prediction accuracy. The present approach provides a guideline to select optimal materials with critical features for the better prediction. More importantly, this quantification approach is potentially applied to deriving a variety of information from any complicated sample such as smells; for example, nanomechanical sensor arrays can correlate various degrees of medical indices with the patterns of biogases such as breath or vapours from sweat, saliva, urine, and tears. Moreover, a target sample of the present approach is not limited to that from gaseous phase but can be also applied to that from liquid phase, leading to the detailed investigation on complex liquid samples such as blood, coffee, and sea water. Therefore, this study paves the way to the tremendous potential applications of nanomechanical sensor arrays through quantitative information extraction in various fields including food, medicine, security, and environment science.

## Methods

### Microfluidic synthesis of silica/titania hybrid nanoparticles with various surface functionalities

#### Chemicals

Triethoxyvinylsilane (TEVS: Tokyo Chemical Industry Co., Ltd.), octadecyltriethoxysilane (ODTES: Tokyo Chemical Industry Co., Ltd.), trimethoxyphenylsilane (TMPS: Tokyo Chemical Industry Co., Ltd.), titanium tetraisopropoxide (TTIP: Tokyo Chemical Industry Co., Ltd.), isopropyl alcohol (IPA: Wako Pure Chemical Industries, Ltd.), 28%-aqueous ammonia solution (NH_3_aq: Kanto Chemical Co., Inc.), octadecylamine (ODA: Aldrich, Inc.), and 3-aminopropyltriethoxysilane (APTES: Sigma, Inc.) were utilized in the present study. All the chemicals were used as received.

### Experimental procedures

Silica/titania hybrid nanoparticles with various surface functionalities were synthesized by means of a multi-step nucleation controlled growth method^17^ which we reported previously with some minor modifications. Briefly, five starting solutions (Solutions A-E) were prepared. Detailed composition of each solution is summarized in Table S1. The Solutions A, B, C, and D were individually flowed in perfluoroalkoxyalkane (PFA: 1.0 mm inner diameter, 1/16 inch outer diameter, product of YMC Co., Ltd.) tubes with a syringe pump (CXN1070, product of ISIS, Co., Ltd.) at 10 mL/min. Solutions A and B, and Solutions C and D were mixed respectively in a polytetrafluoroethylene (PTFE) fluidic channel with a Y shape junction (the channel cross section of ca. 1 mm^2^, KeyChem mixer, product of YMC Co., Ltd.). After that, resultant two reaction solutions, *i*.*e*. Solution A + B and Solution C + D, were mixed in the second fluidic channel placed just after the first two fluidic channels. The first and second fluidic channels were connected by 10 cm PFA tubes. Then, the mixture of the four Solutions A-D was flowed through a PFA tube with 70 cm in length and was added into the Solution E under magnetic stirring. After the addition, the final reaction solution was aged at room temperature for 24 h. Finally, a slightly turbid suspension was obtained.

### Characterization

Fourier Transform-Infrared (FT-IR) spectra were measured using a Nicolet 4700 FT-IR spectrometer (Thermo Fisher Scientific Inc.) at the resolution of 2.0 cm^−1^. The sample powder was homogeneously mixed with KBr, and then the mixture was pressed to form a KBr disk for the transmission measurements. Scanning electron microscope (SEM) images were obtained using a Hitachi Ultra-high Resolution Scanning Electron Microscope SU8000 at the accelerating voltage of 10 kV. Prior to each measurement, samples were coated with a few nanometers of platinum.

### Spray coating of various nanoparticles onto MSS

The detailed fabrication procedure of the MSS chip was described in our previous reports. Each of the four nanoparticle suspensions was spray-coated onto the surface of MSS by using a spray coater (rCoater, product of Asahi Sunac Co.). For the preparation of the nanoparticle suspensions, all the functional nanoparticles were centrifuged at 9000 rpm for 10 min. The sediment was carefully washed with IPA several times and then IPA/water mixture (vol/vol = 3/5) was added. The concentration of the four suspensions was set at approx. 1 g/L. Before spray-coating, the suspensions were fully ultrasonicated to get the nanoparticles dispersed as much as possible (some aggregates were still recognized).

Then, the suspension was loaded in a syringe and was flowed through a PTFE tube at 3 mL/min by using a syringe pump (YSP-201, product of YMC Co., Ltd.). The suspension was introduced into a spray nozzle and then was sprayed with the help of two types of carrier air (atomizing air: 0.030 MPa, patterning air: 0.030 MPa) to form homogeneous droplets. The MSS was mounted on a stage which was heated at approx. 100 °C to quickly evaporate the droplets. The stage was moved back and forth, while the spray nozzle was also moved from left to right at 15 mm/sec with 0.3 mm pitch. The distance between the spray nozzle and stage was set at 100 mm. The coating process was repeated to obtain coating thickness of around 1 μm. To avoid any cross contamination, a mask was used to cover three channels when one channel was in a coating process.

### Sensing experiments

#### Sample liquids

Formaldehyde solution (35–38%), *n*-hexane, ethanol, isopropyl alcohol, benzene, toluene, xylene, 2-butanone, and acetic acid were purchased from Wako Pure Chemical Industries, Ltd. Methanol and ethyl acetate were purchased from Kanto Chemical Co., Inc. *n*-heptane and *n*-octane were purchased from Nacalai Tesque, Inc. Acetone was purchased from Sigma-Aldrich Co. All the chemicals were used as received.

For the alcohol content quantification under an ambient condition, following samples were used (alcohol content of each sample is shown in parentheses):

Ultrapure water (0%), bottled water (0%), tap water (0%), phosphate buffered saline (PBS; 0%), green tea (0%), oolong tea (0%), shochu and green tea (4%), beer (5%), shochu and oolong tea (6%), sangria (9%), ume-shu (plum wine; 12%), red wine (12%), junmai ryori-shu (Japanese cooking wine; 14%), mirin (a type of rice wine; 14.5%), Japanese sake (15%), shoko-shu (Shaoxing rice wine; 17.5%), mugi-shochu (a Japanese distilled beverage distilled from barley; 20%), cassis-flavored liqueur (20%), plant worm-shochu (a Japanese distilled beverage distilled from plant worm; 25%), imo-shochu (a Japanese distilled beverage distilled from sweet potatoes; 25%), vodka (40%), gin (40%), palinka (40%), rum (40%), brandy (40%), and whisky (40%).

In addition, following water/EtOH mixed solutions with different composition were also used:

Water/EtOH volume ratio of 95/5, 90/10, 85/15, 80/20, 75/25, 70/30, 65/35, 60/40, and 55/45.

### Detailed procedure and conditions for the sensing experiments

In the present study, the MSS functionalized with various NPs was mounted on a chamber and the chamber was carefully sealed with O-rings. Two piezoelectric pumps were utilized to introduce atmospheric air into the chamber at the flow rate of 14 mL/min. One pump was for purging (*i*.*e*. accelerating desorption of adsorbents), and the other one was for introducing sample vapour together with air. In the present case, a given amount of sample liquid was added into a small vial capped with a rubber lid and a needle connected to a PTFE tube was stuck into the headspace of the vial through the rubber lid. The other end of the PTFE tube was connected to the piezoelectric pump to allow for the sample vapour collection from the headspace. Please note that another needle that was connected to a PTFE tube and the other end of the tube was open to air was also stuck into the headspace of the vial to let the fluid flow smooth. The two piezoelectric pumps were switched every 10 seconds to perform a sample introduction-purging cycle. This cycle was repeated five times and the data were recorded at the bridge voltage of −1.0 V and sampling rate of 20 Hz. All the experiments were conducted under an ambient condition without any temperature/humidity control.

### Kernel ridge regression

Kernel ridge regression (KRR)^24^ is one of the powerful machine learning techniques to predict unknown data from a known dataset. Let us consider the case that *N* data ${\{{{\bf{X}}}_{n},A({{\bf{X}}}_{n})\}}_{n=1,\ldots ,N}$ are given. In the present study, **X**_*n*_ is the vector where its elements are the parameters extracted from a response signal of the smell of a liquid sample labelled by *n*. Here, each extracted parameter is normalized by the standard deviation of *n* data points. Furthermore, *A*(**X**_*n*_) denotes the alcohol content of its liquid sample. Note that the dimension of **X**_*n*_ depends on the numbers of used parameters and channels of the MSS. In this case, by KRR, the alcohol content A*(**X**) of an unknown liquor with **X**, which is obtained by a response signal of the smell of the unknown liquor, is predicted as

5 $${A}^{\ast }({\bf{X}})={{\bf{k}}}^{{\rm{T}}}{({\bf{K}}+\lambda {\bf{I}})}^{-1}{\bf{A}},$$

where

6 $${\bf{A}}={(A({{\bf{X}}}_{1})\cdots A({{\bf{X}}}_{N}))}^{{\rm{T}}},$$

7 $${\bf{k}}={(k({{\bf{X}}}_{1},{\bf{X}})\cdots k({{\bf{X}}}_{N},{\bf{X}}))}^{{\rm{T}}},$$

8 $${\bf{K}}=(\begin{array}{ccc}k({{\bf{X}}}_{1},{{\bf{X}}}_{1}) & \cdots  & k({{\bf{X}}}_{1},{{\bf{X}}}_{N})\\ \vdots  & \ddots  & \vdots \\ k({{\bf{X}}}_{N},{{\bf{X}}}_{1}) & \cdots  & k({{\bf{X}}}_{N},{{\bf{X}}}_{N})\end{array}).$$

Here, **I** is the *N* × *N* unit vector and $k({{\bf{X}}}_{n},{{\bf{X}}}_{m})$ is the kernel function which represents the similarity between ${{\bf{X}}}_{n}$ and ${{\bf{X}}}_{m}$, and we used the Gaussian kernel:

9 $$k({{\bf{X}}}_{n},{{\bf{X}}}_{m})=\exp [-\frac{1}{2{\sigma }^{2}}{|{{\bf{X}}}_{n}-{{\bf{X}}}_{m}|}^{2}].$$

In this procedure, *λ* and *σ* are the hyperparameters which should be given before the analysis. The prediction performance strongly depends on values of the hyperparameters. Notice that various regression methods exist in machine learning, and then our approach in the present study is just one example.

### Cross validation

To decide values of the hyperparameters *λ* and *σ* in KRR, we used the cross validation^24^. In the cross validation, some data are removed from the dataset before training, and the removed data are regarded as the testing data which are used to validate a prediction. We estimated the cross-validation error expressing the prediction performance, and the values of the hyperparameters are evaluated so that the cross-validation error is minimized. In the following, the detailed procedure of *S*-fold cross validation is shown.

First, the dataset *D* is randomly divided into *S* data subsets. Each data subset is expressed by *D*_*s*_ which is labeled by *s* = 1,…, *S*, and the number of data in each data subset is $N/S$. One of the *S* data subsets is regarded as the testing dataset, while the remaining $S-1$ data subsets are used as the training dataset. The number of testing data and that of training data are given by ${N}_{{\rm{te}}}=N/S$ and ${N}_{{\rm{tr}}}=N(S-1)/S$, respectively. Next, for each data subset ${G}_{s}=D\setminus {D}_{s}$ consisting of *N*_tr_ data, we perform KRR for various *λ* and *σ*, and the predicted alcohol content of a liquor with **X**, ${A}^{\ast (s)}({\bf{X}};\,\lambda ,\,\sigma )$, is obtained depending on *λ* and *σ*. We calculate the mean-square deviation between the alcohol contents of testing dataset *D*_*s*_ and predicted alcohol contents:

10 $${{\rm{\Delta }}}^{(s)}(\lambda ,\,\sigma )=\frac{1}{{N}_{{\rm{t}}{\rm{e}}}}\sum _{l\,\in {D}_{s}}{[A({{\bf{X}}}_{l})-{A}^{\ast (s)}({{\bf{X}}}_{l};\lambda ,\sigma )]}^{2}.$$

Furthermore, the cross-validation error depending on *λ* and *σ* is obtained by averaging *S* different mean-square deviations, which is defined as

11 $${\rm{\Delta }}(\lambda ,\,\sigma )=\frac{1}{S}\sum _{s=1}^{S}{{\rm{\Delta }}}^{(s)}(\lambda ,\,\sigma ).$$

By minimizing Δ(*λ*, *σ*) with respect to *λ* and *σ*, we evaluate the optimal values of the hyperparameters λ* and σ*, which realize a good prediction. Finally, we define the prediction error of each trained ML model which expresses a prediction performance as

12 $${\rm{\Delta }}={\rm{\Delta }}({\lambda }^{\ast },\,{\sigma }^{\ast }).$$

By using the prediction error Δ, we search the optimal combination of parameters extracted from a response signal and the optimal receptor layer materials to predict the alcohol content of liquors in the present study. Notice that various methods to decide the hyperparameters in a ML model exist and there are also various definitions of the prediction error, and then the machine learning approach we adopted is just one example.

## Electronic supplementary material

Supplementary information
