# Supplementary material for: Data-driven nanomechanical sensing: specific information extraction from a complex system
Source: Sci Rep. 2017 Jun 16;7:3661. doi: 10.1038/s41598-017-03875-7 (PMC5473933; doi:10.1038/s41598-017-03875-7)
Supplement: Supplementary file 1 — Supplementary information [file 41598_2017_3875_MOESM1_ESM.docx]

**Data-driven nanomechanical sensing: specific information extraction from a complex system**

Kota Shiba^1^*, Ryo Tamura^1,2^*, Gaku Imamura^1,2,3^, and Genki Yoshikawa^1,4^

^1^World Premier International Research Center Initiative (WPI), International Center for Materials Nanoarchitectonics (MANA), National Institute for Materials Science (NIMS), 1-1 Namiki, Tsukuba, Ibaraki 305-0044, Japan

^2^Center for Materials Research by Information Integration (CMI^2^), National Institute for Materials Science (NIMS), 1-2-1 Sengen, Tsukuba, Ibaraki 305-0047, Japan

^3^International Center for Young Scientists (ICYS), National Institute for Materials Science (NIMS), 1-1 Namiki, Tsukuba, Ibaraki 305-0044, Japan

^4^Materials Science and Engineering, Graduate School of Pure and Applied Science, University of Tsukuba, Tennodai 1-1-1 Tsukuba, Ibaraki 305-8571, Japan

*Corresponding authors

**Kota Shiba**

E-mail: SHIBA.Kota@nims.go.jp

Tel: +81-29-860-4603, Fax: +81-29-860-4706

**Ryo Tamura**

E-mail: TAMURA.Ryo@nims.go.jp

Tel: +81-29-860-4948, Fax: +81-29-860-4706

**Supplementary Figures and Tables**

**
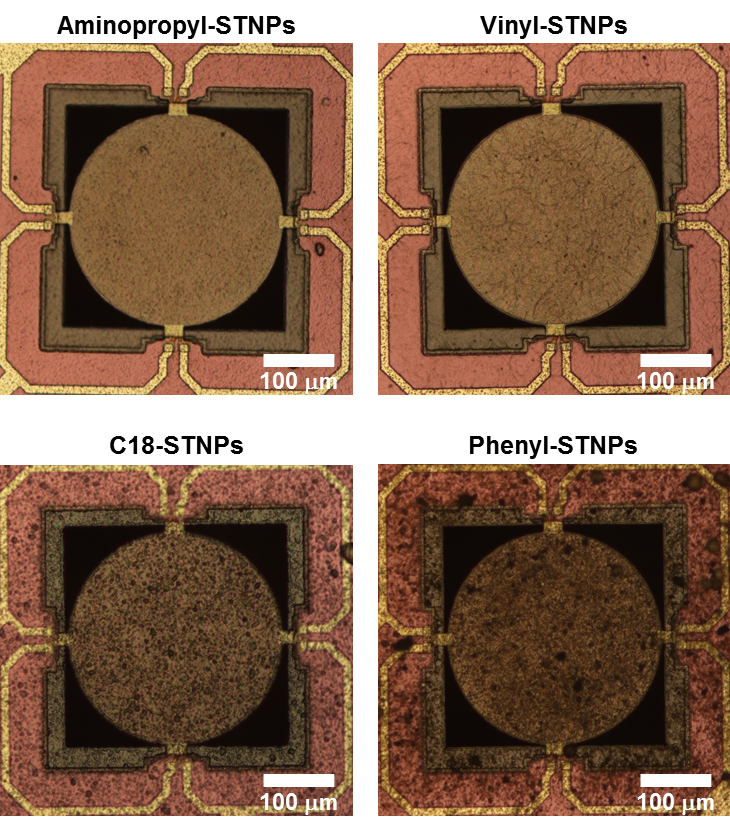
**

**Figure S1** Optical microscope images of the Aminopropyl-STNPs, Vinyl-STNPs, C18-STNPs, and Phenyl-STNPs coated MSS.

**Aminopropyl-STNPs**

**
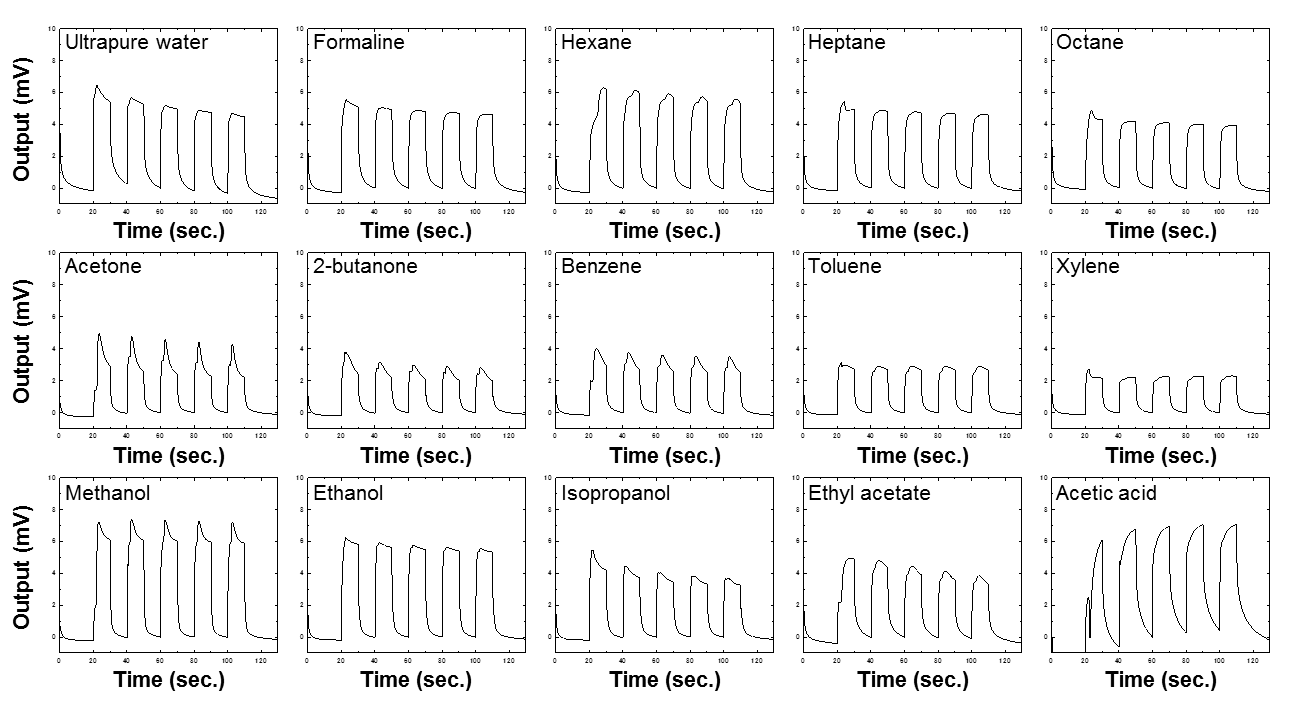
**

**Vinyl-STNPs**

**
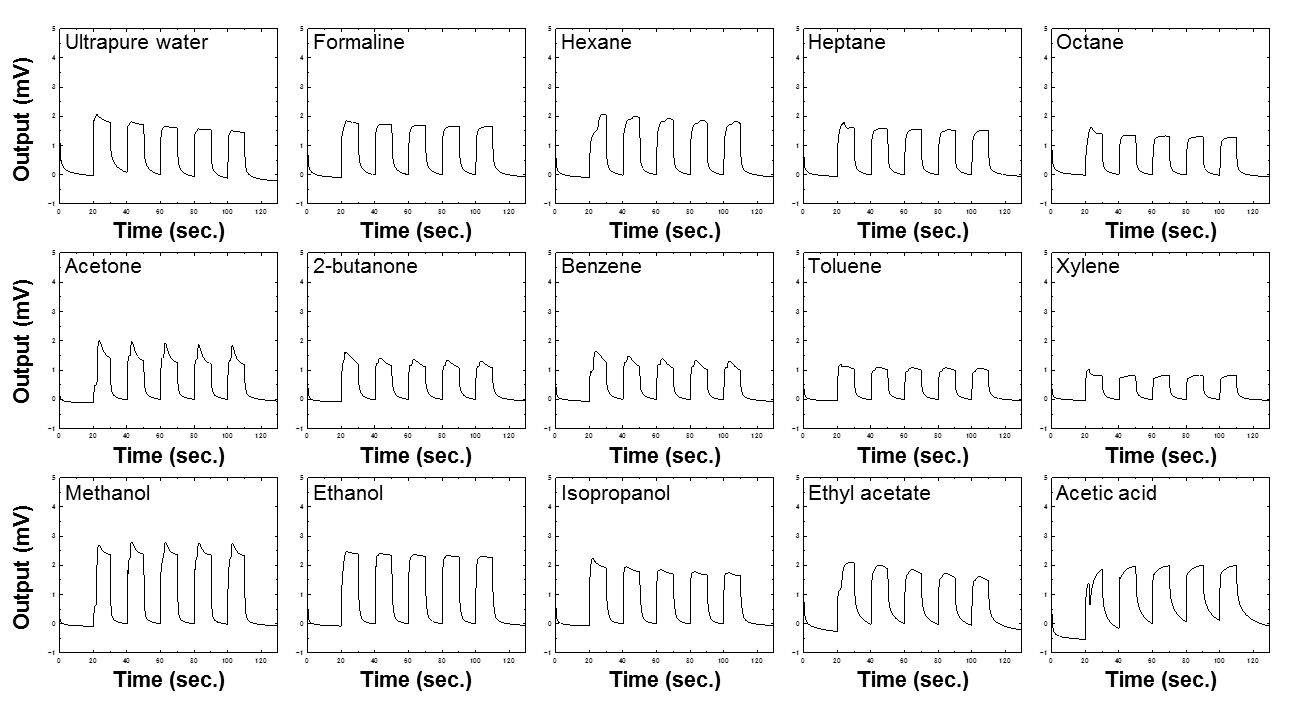
**

**C18-STNPs**

**
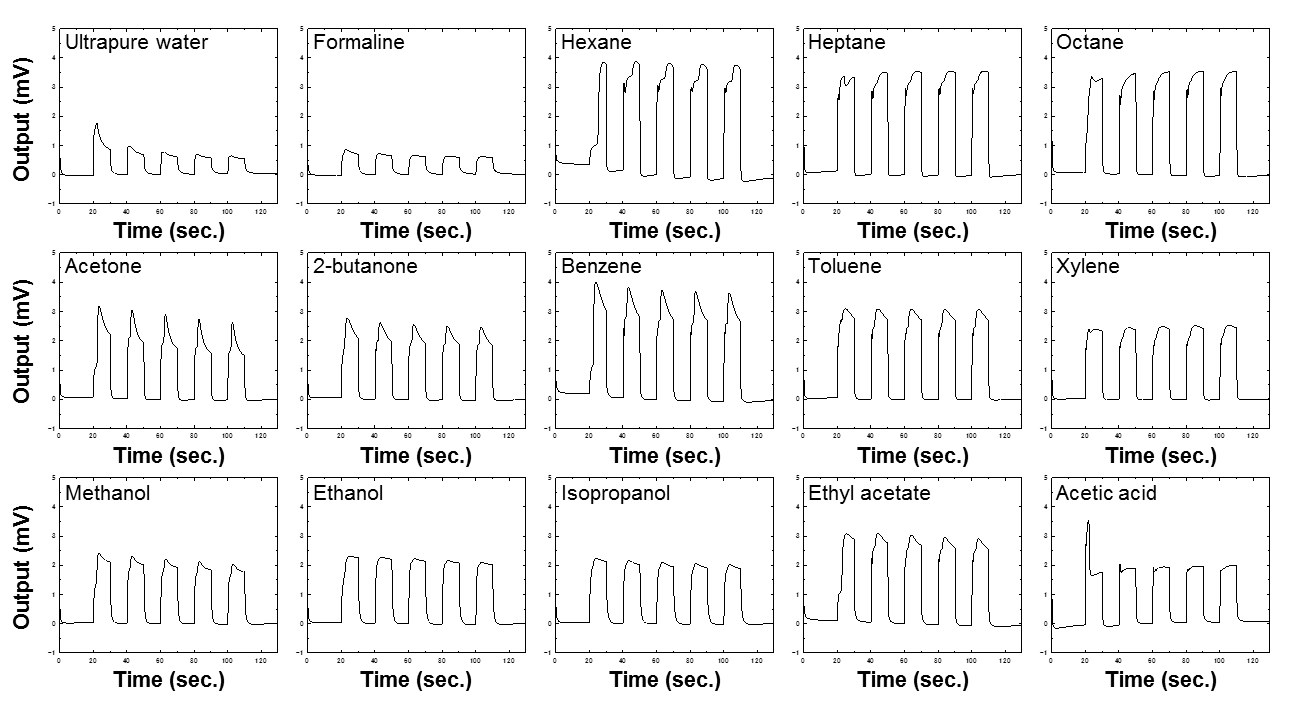
**

**Phenyl-STNPs**

**
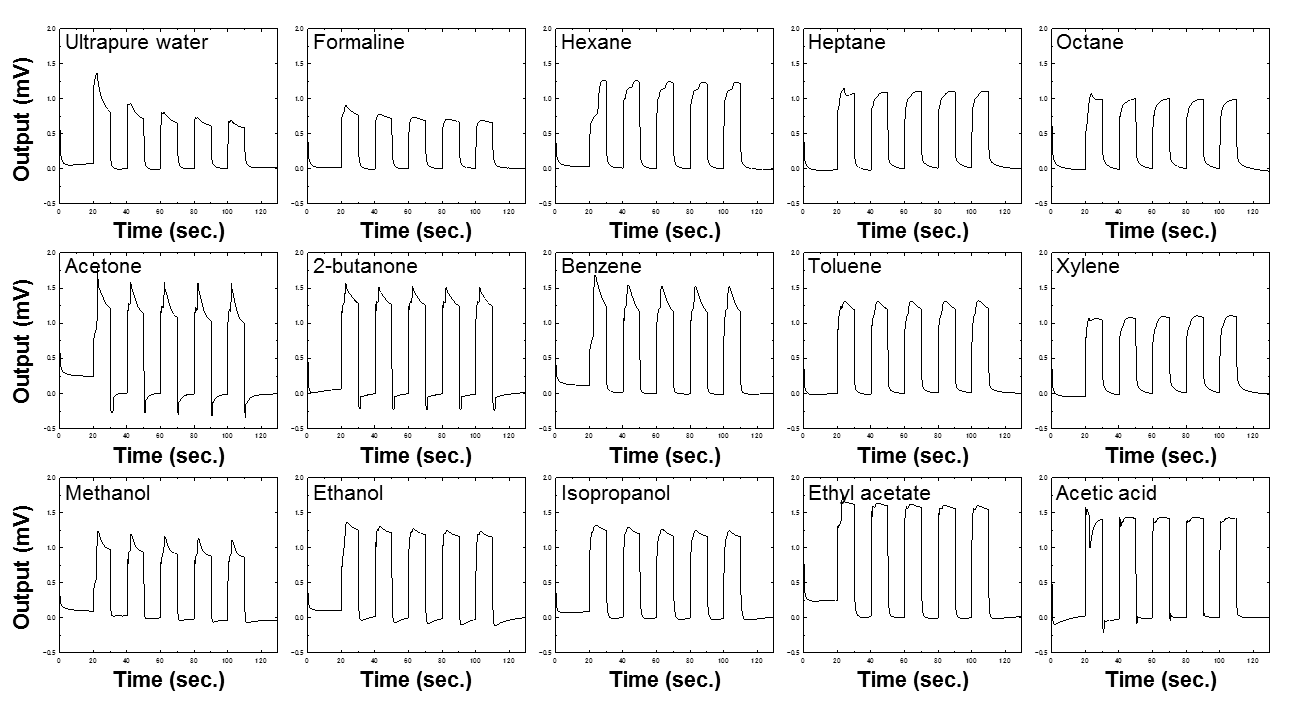
**

**Figure S2** Responses of the Aminopropyl-STNPs, Vinyl-STNPs, C18-STNPs, and Phenyl-STNPs coated MSS to 15 chemicals under an ambient condition. The names of the measured chemicals are shown in each graph.

**Aminopropyl-STNPs**

**
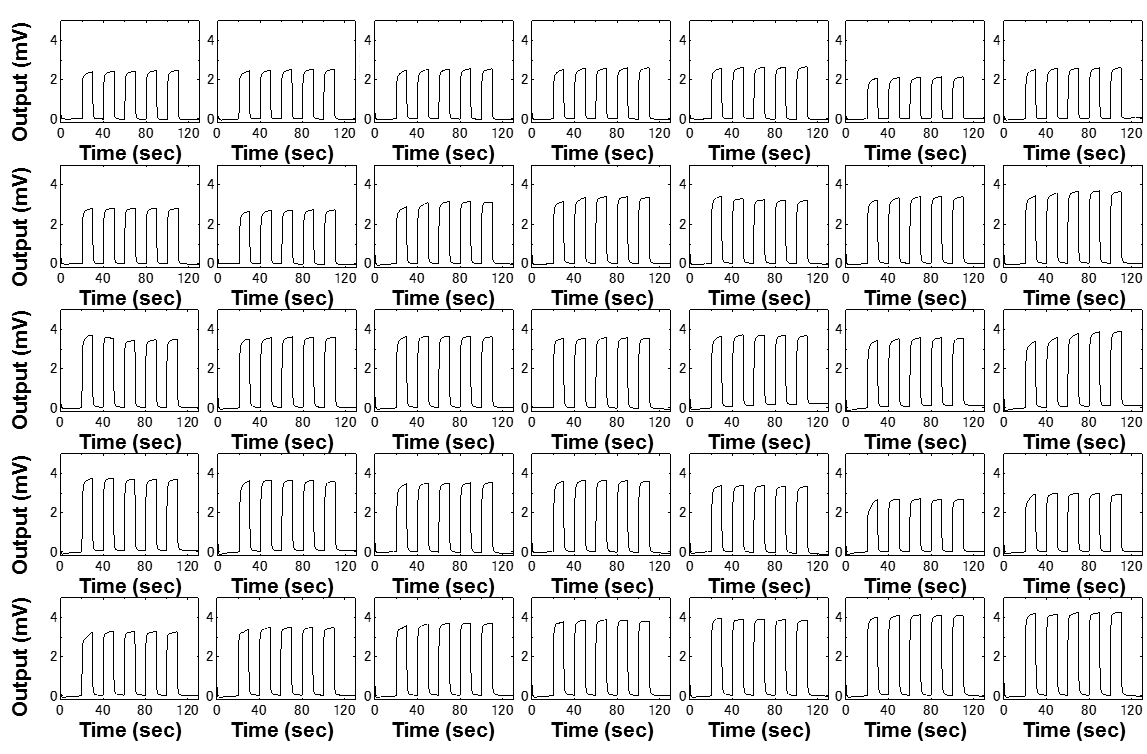
**

**Vinyl-STNPs**

**
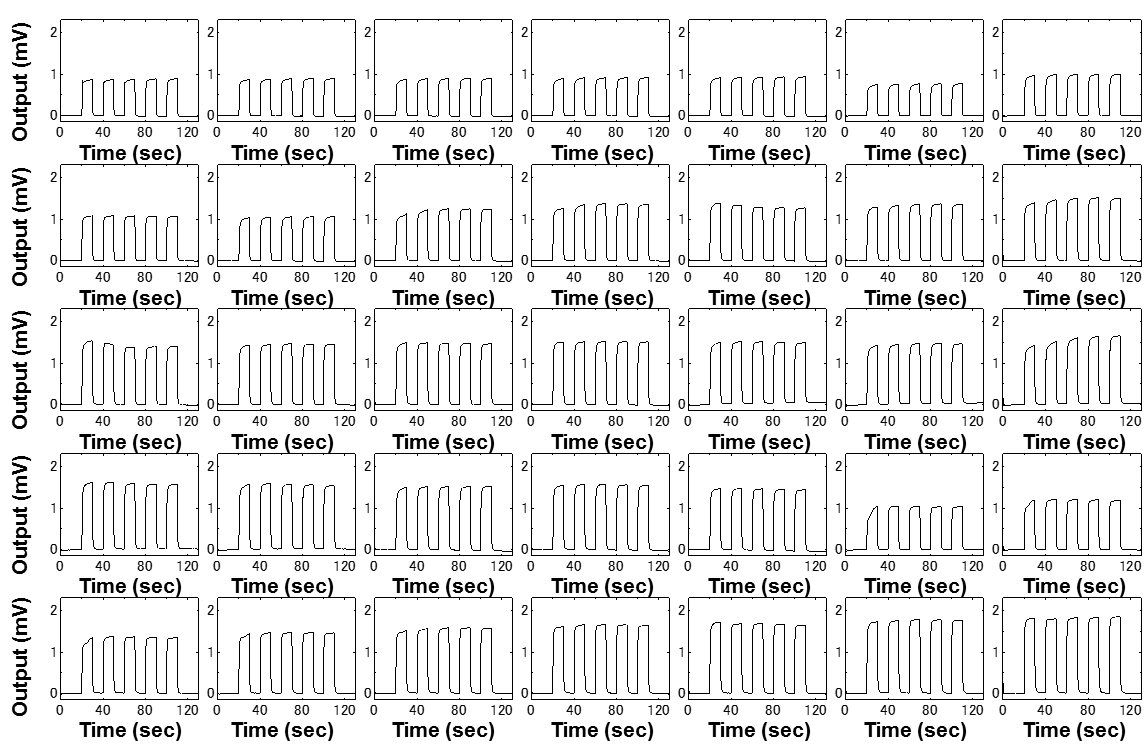
**

**C18-STNPs**

**
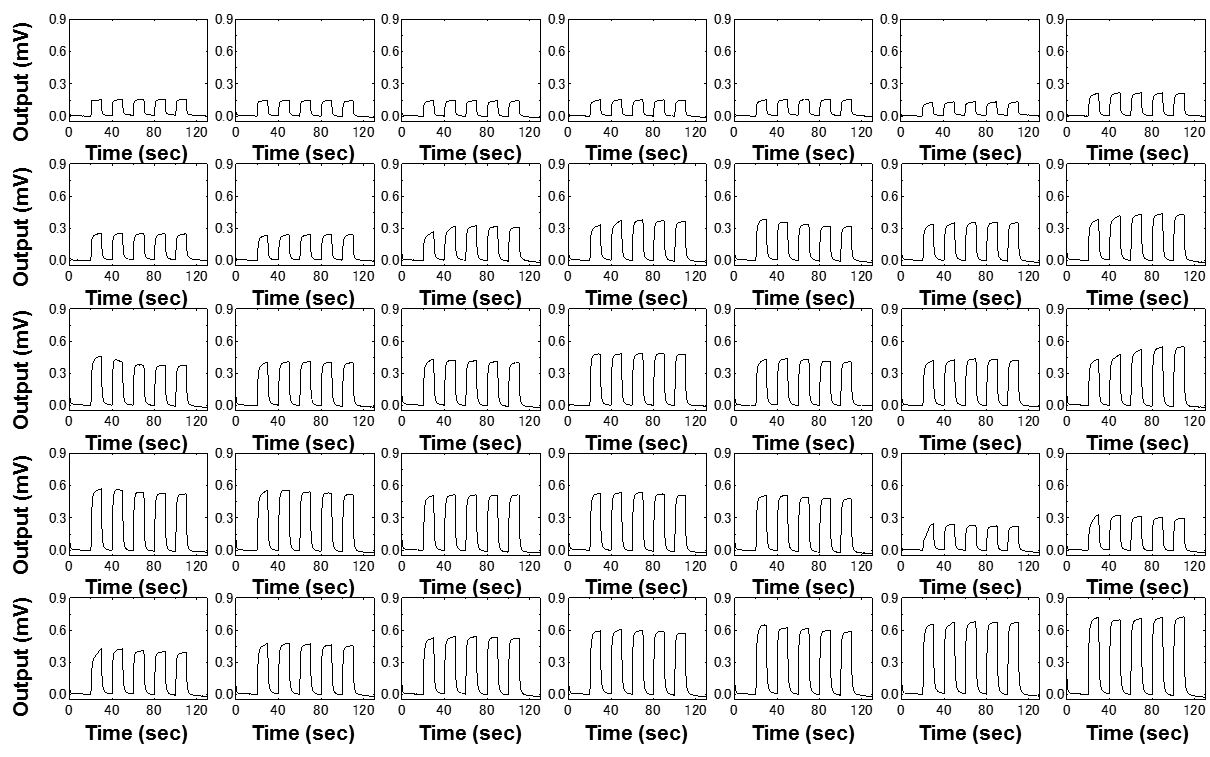
**

**Phenyl-STNPs**

**
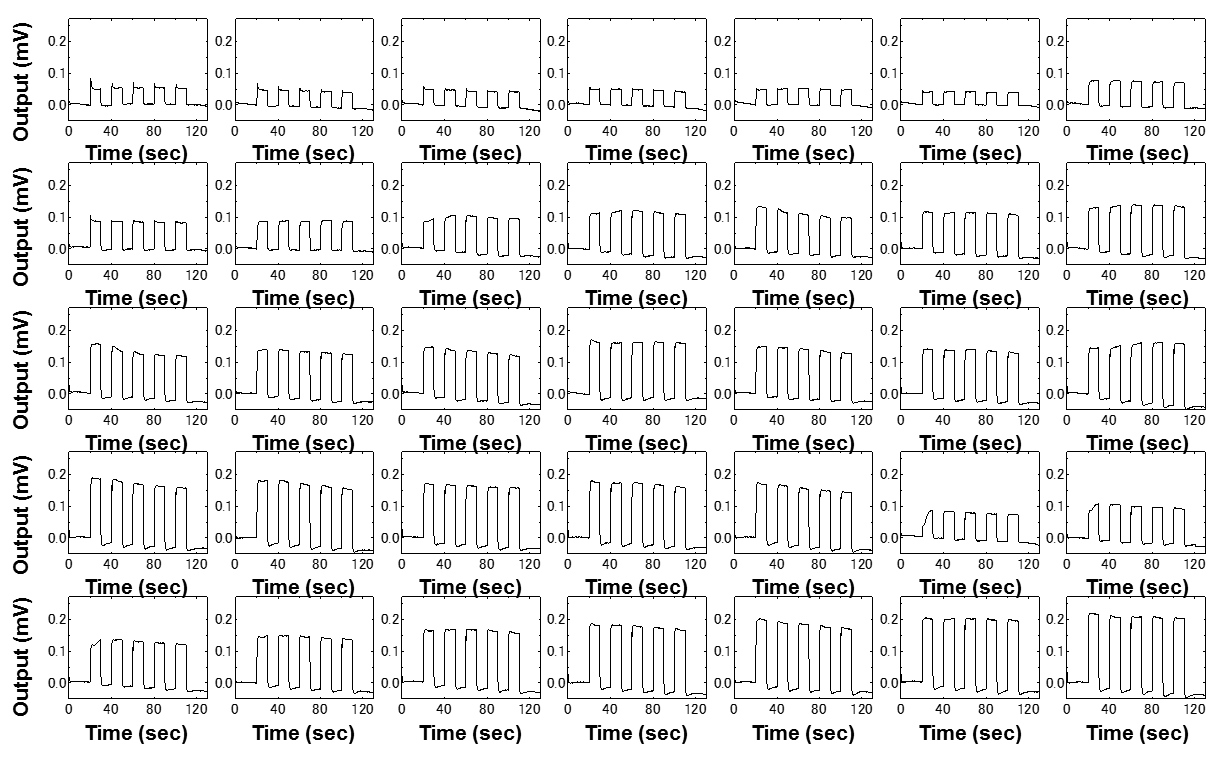
**

**
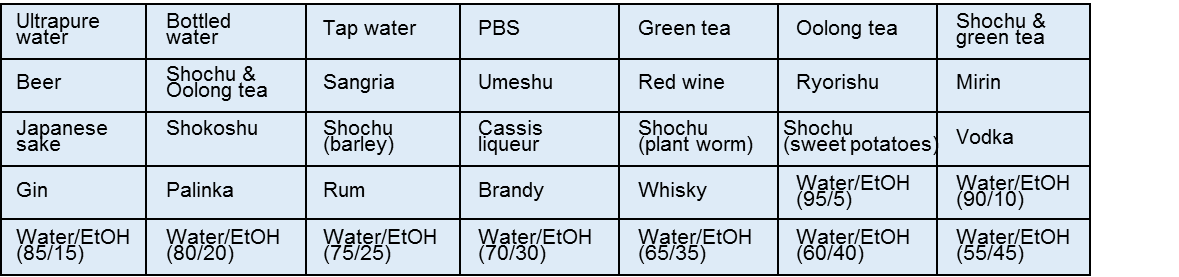
**

**Figure S3** Responses of the Aminopropyl-STNPs, Vinyl-STNPs, C18-STNPs, and Phenyl-STNPs coated MSS to 35 liquid samples including waters, teas, alcohols, and aqueous EtOH with different compositions under an ambient condition. The names of the measured samples are summarized in the box and the position of the names corresponds to those shown in a bunch of graphs.

**Aminopropyl-STNPs**

**
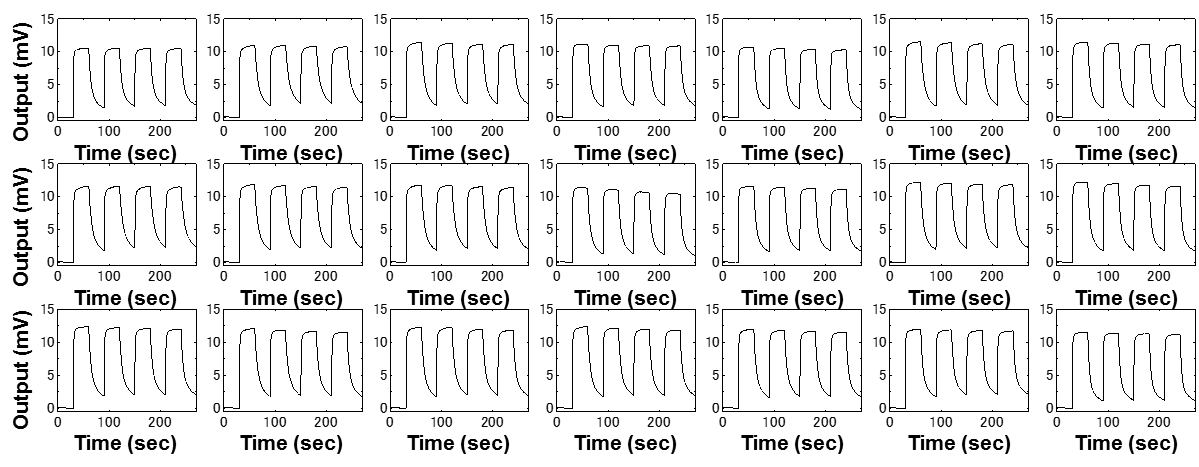
**

**Vinyl-STNPs**

**
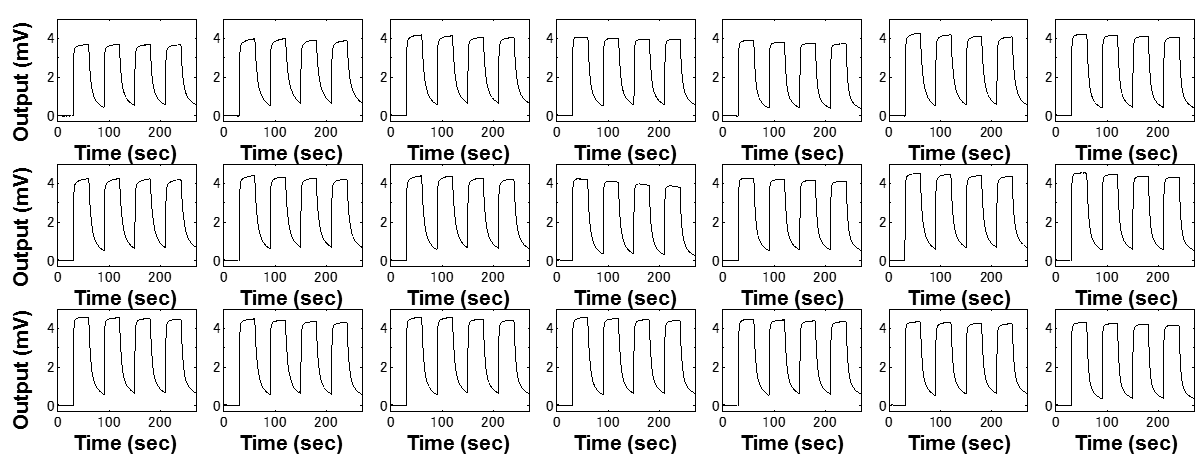
**

**C18-STNPs**

**
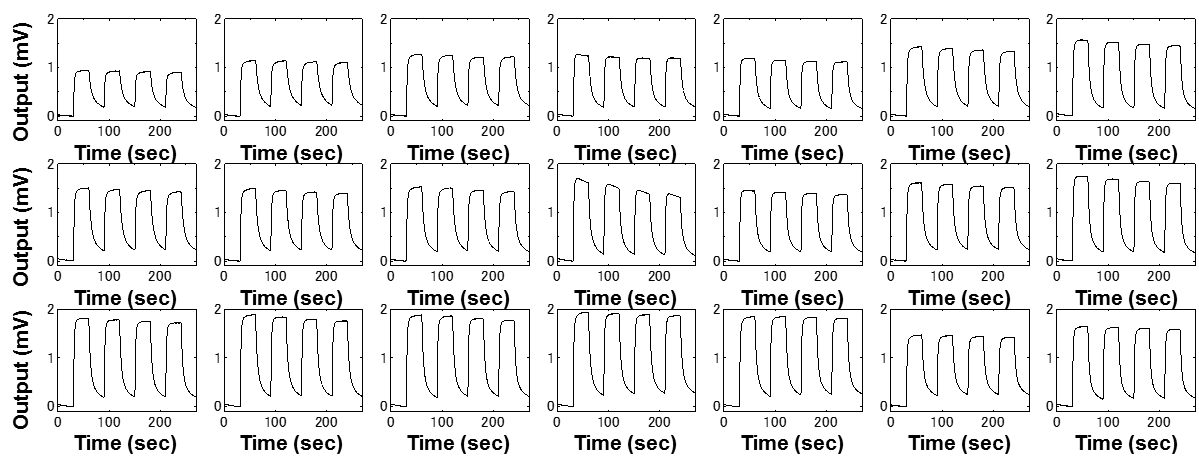
**

**Phenyl-STNPs**

**
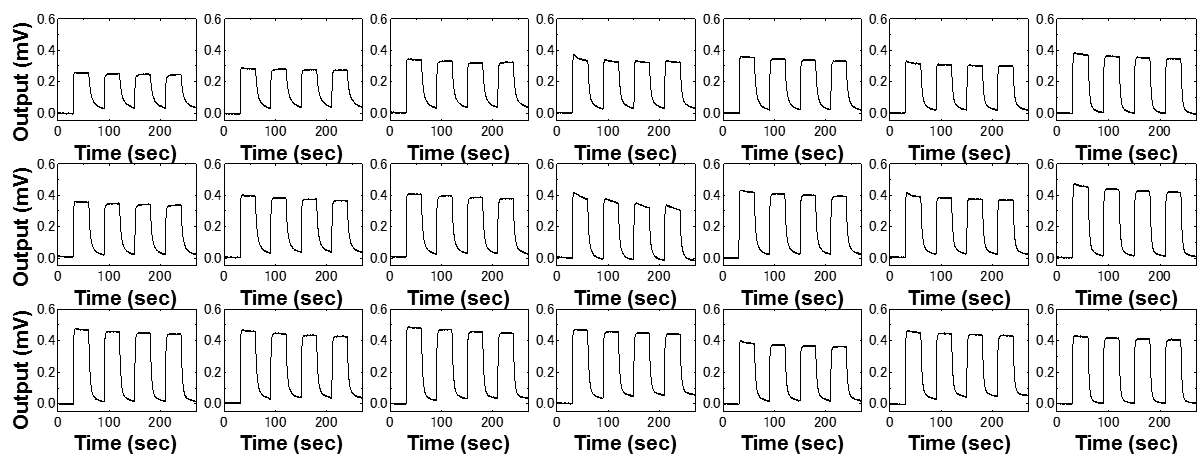
**

**
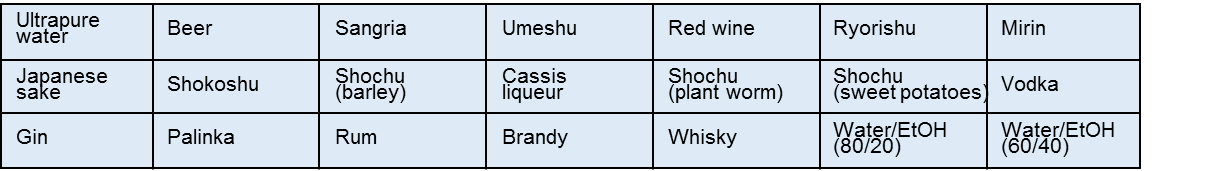
**

**Figure S4** Responses of the Aminopropyl-STNPs, Vinyl-STNPs, C18-STNPs, and Phenyl-STNPs coated MSS to 21 liquid samples including water, alcohols, and aqueous EtOH with different compositions under an N_2_ condition. The names of the measured samples are summarized in the box and the position of the names corresponds to those shown in a bunch of graphs.

**Polysulfone**


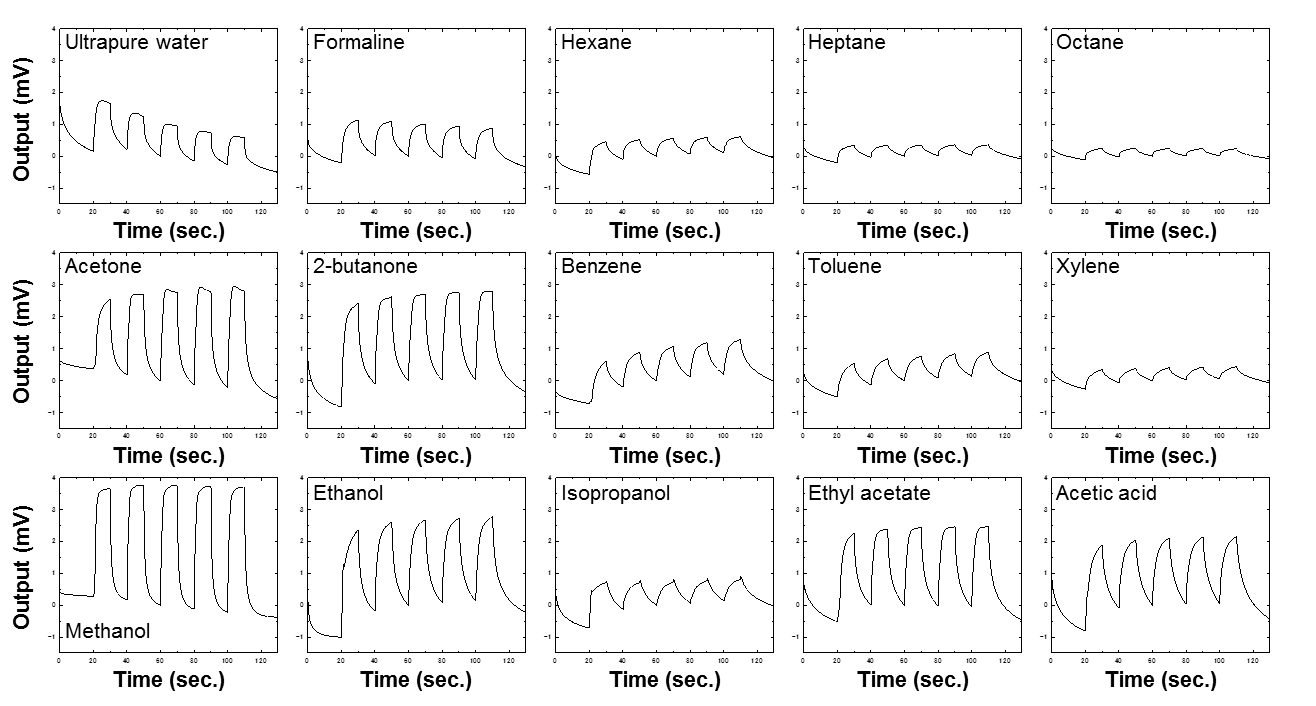


**Polycaprolactone**


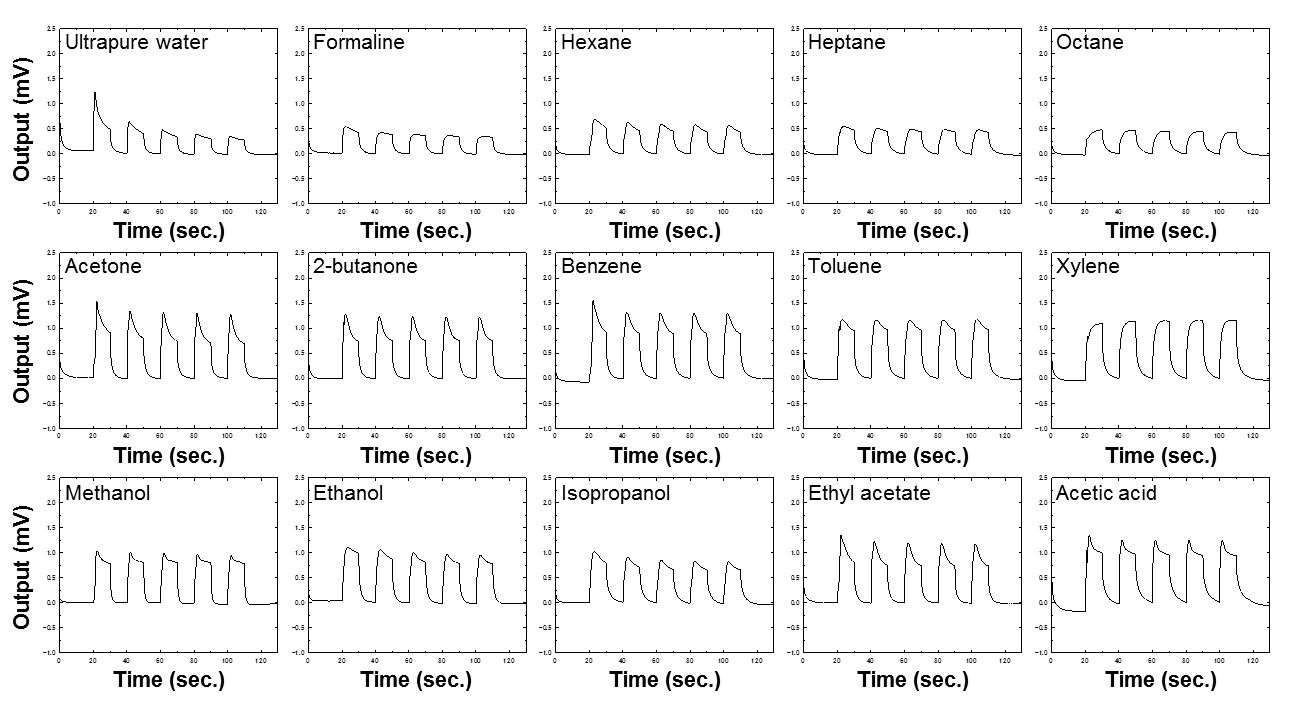


**Figure S5** Responses of the polysulfone and polycaprolactone coated MSS to 15 chemicals under an ambient condition. The names of the measured chemicals are shown in each graph.

**Polysulfone**

**
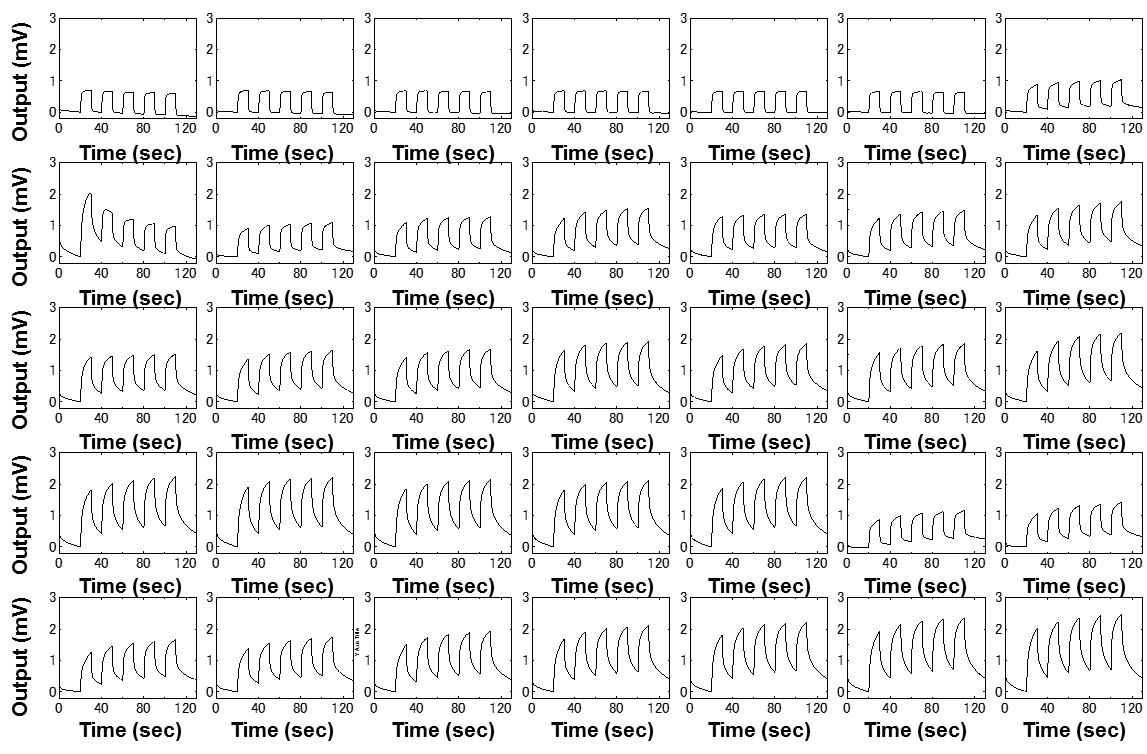
**

**Polycaprolactone**

**
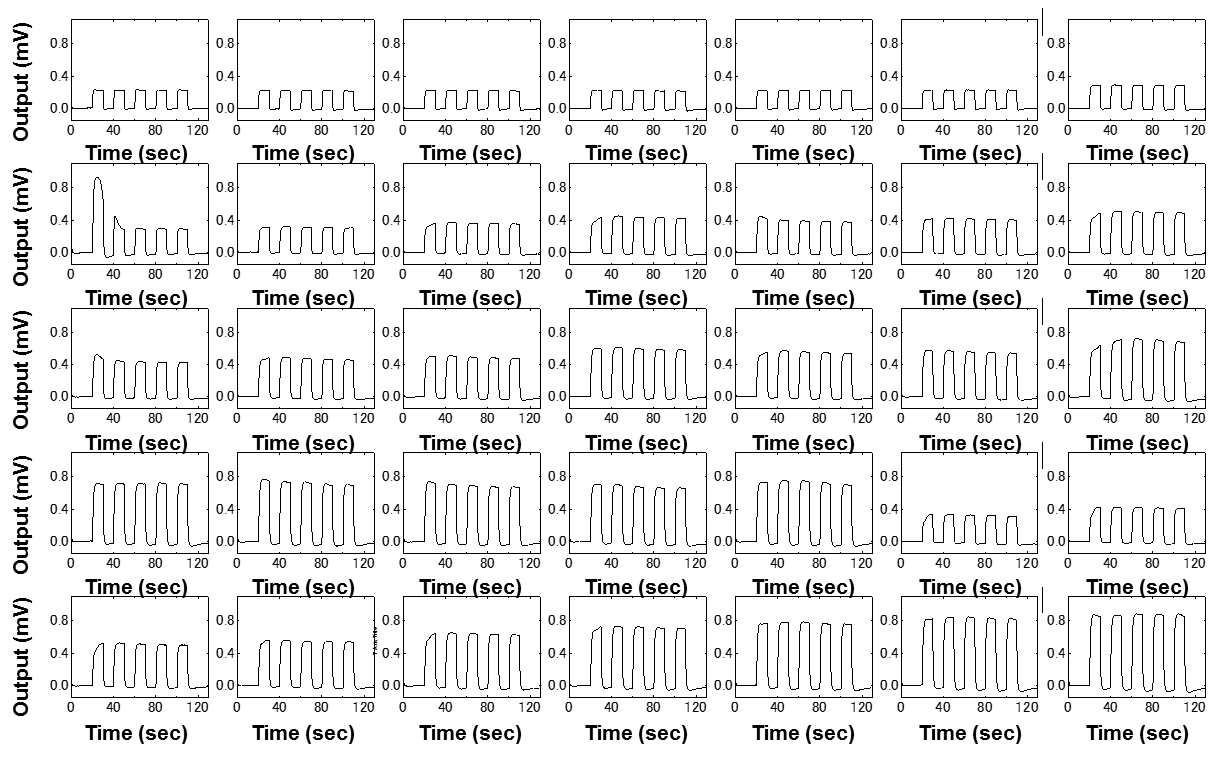
**

**
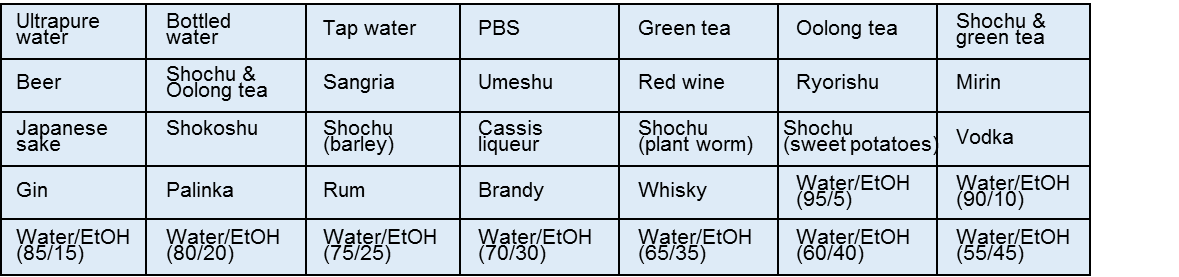
Figure S6** Responses of the polysulfone and polycaprolactone coated MSS to 35 liquid samples including waters, teas, alcohols, and aqueous EtOH with different compositions under an ambient condition. The names of the measured samples are summarized in the box and the position of the names correspond to those shown in a bunch of graphs.

**Polysulfone**

**
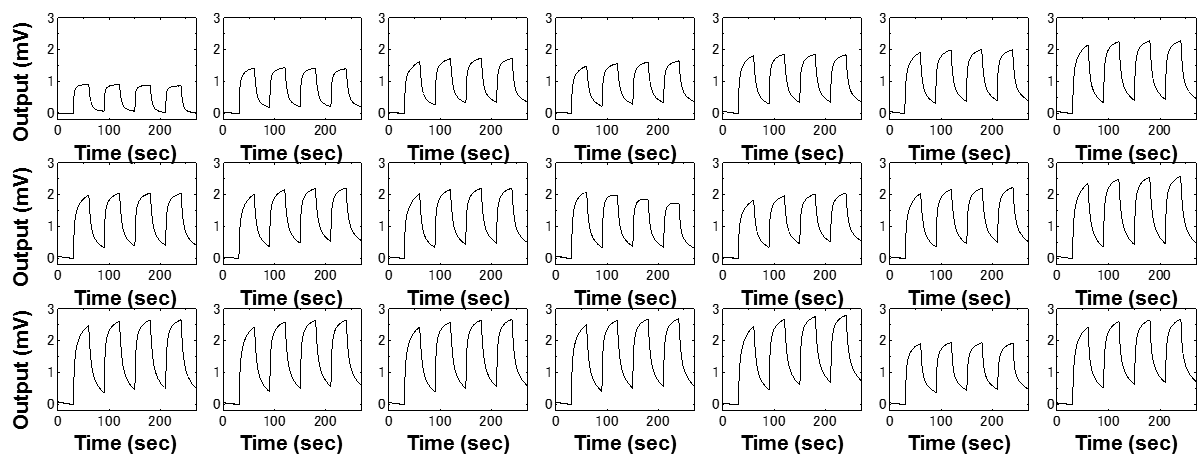
**

**Polycaprolactone**

**
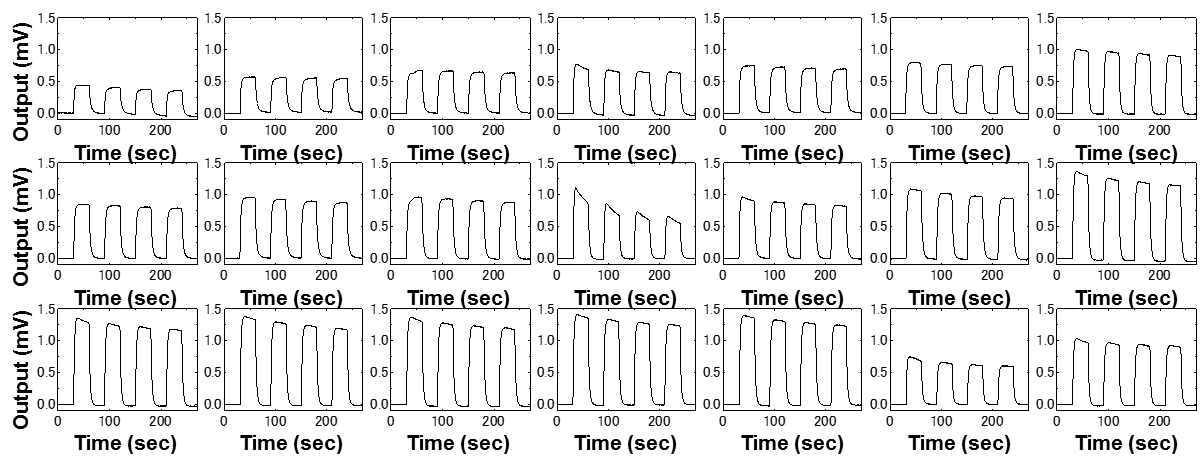
**

**
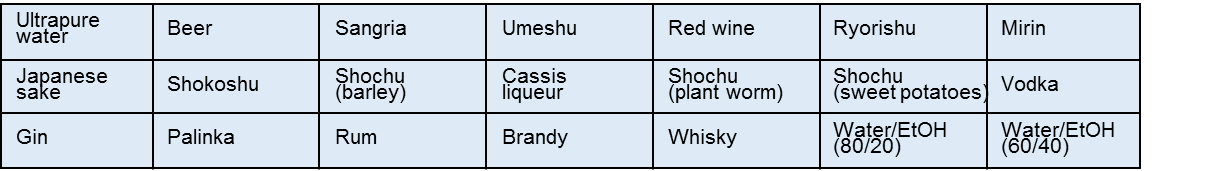
**

**Figure S7** Responses of the polysulfone and polycaprolactone coated MSS to 21 liquid samples including water, alcohols, and aqueous EtOH with different compositions under an N_2_ condition. The names of the measured samples are summarized in the box and the position of the names corresponds to those shown in a bunch of graphs.

**Table S1** The amount of each chemical used for the synthesis of various STNPs.

| Solution A | | Solution B | | | Solution C | | Solution D | | Solution E | | |
| --- | --- | --- | --- | --- | --- | --- | --- | --- | --- | --- | --- |
| **※1**  (mL) | IPA  (g) | NH_3_aq  (g) | H_2_O  (g) | IPA  (g) | TTIP  (mL) | IPA  (g) | H_2_O  (mL) | IPA  (g) | ODA  (g) | H_2_O  (mL) | IPA  (g) |
| **※2** | **※3** | 0.758 | 2.84 | 6.98 | 0.458 | 9.44 | 0.078 | 9.74 | 0.1368 | 40 | 123.3 |

**※1**

Aminopropyl-STNPs -> APTES

Vinyl-STNPs -> TEVS

C18-STNPs -> ODTES

Phenyl-STNPs -> TMPS

**※2**

Aminopropyl-STNPs -> 1.481

Vinyl-STNPs -> 1.330

C18-STNPs -> 2.000

Phenyl-STNPs -> 1.160

**※3**

Aminopropyl-STNPs -> 8.639

Vinyl-STNPs -> 8.757

C18-STNPs -> 8.232

Phenyl-STNPs -> 8.890

**Supplementary Note**

1. **Training results under an ambient condition by Polymers**

**Figure A-1** is the alcohol content dependence of the parameters extracted from the response signals of the 35 liquid samples measured by the MSS (samples are described in Method section 3-1) when Polysulfone and Polycaprolactone were used as a receptor layer material. Moderate correlations of parameters with respect to the alcohol content were confirmed for all parameters. **Figure A-2** and **Table A-1** are the training results under an ambient condition when Polysulfone and Polycaprolactone were used as a receptor layer material. The setting is completely the same with the NPs cases. For the known liquid samples, the prediction by the ML model was successful for both cases.

**
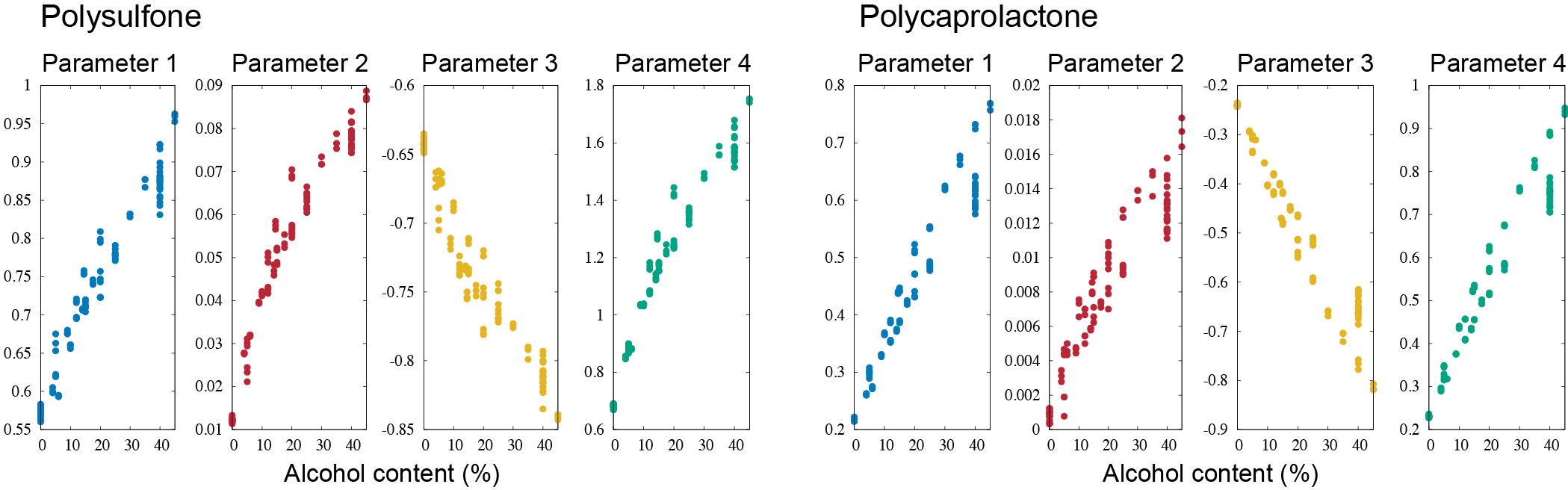
**

**Fig. A-1** Alcohol content dependence of the parameters extracted from response signals under an ambient condition. In each case, the 105 data exist.


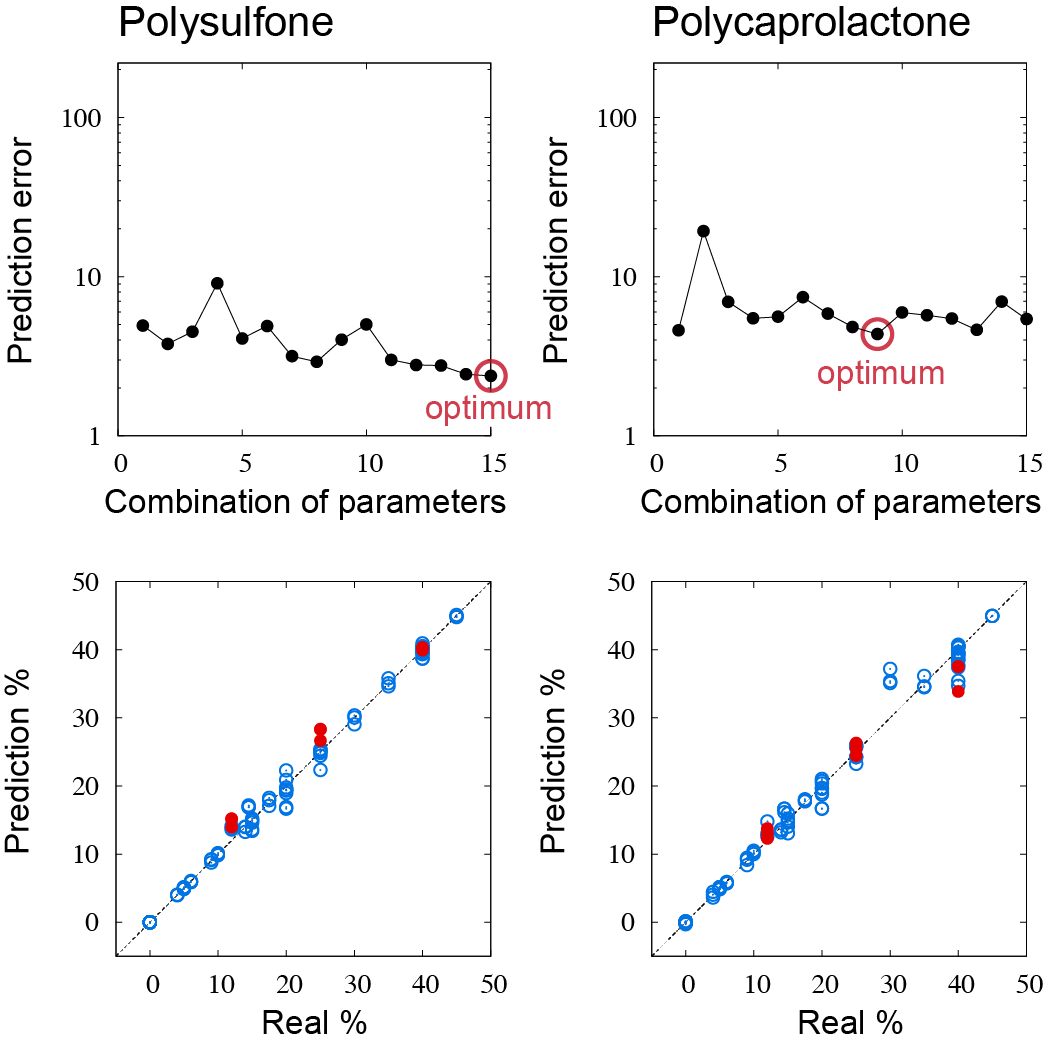


**Fig. A-2** (Top) Prediction errors depending on the combination of four parameters extracted from a response signal under an ambient condition. The definition of combinations by the decimal number was explained in caption of **Fig. 8**. (Bottom) Parity plot of predicted alcohol content versus real alcohol content under an ambient condition. The blue points represent the known liquid samples which are used to train a ML model. The red points are the unknown liquors: red wine (12%), imo-shochu (25%), and whisky (40%).

**Table A-1** Optimal combination of parameters and optimal prediction error depending on the receptor layer material under an ambient condition.

|  | Polysulfone | Polycaprolactone |
| --- | --- | --- |
| Parameter 1 | Use | Use |
| Parameter 2 | Use |  |
| Parameter 3 | Use |  |
| Parameter 4 | Use | Use |
| Prediction error | 2.3757 | 4.3535 |

1. **Training results under an N_2_ environment**

**B-1 Sample liquids**

For the alcohol content quantification, following samples were used (alcohol content of each sample is shown in parentheses):

Ultrapure water (0%), beer (5%), sangria (9%), ume-shu (plum wine; 12%), red wine (12%), junmai ryori-shu (Japanese cooking wine; 14%), mirin (a type of rice wine; 14.5%), Japanese sake (15%), shoko-shu (Shaoxing rice wine; 17.5%), mugi-shochu (a Japanese distilled beverage distilled from barley; 20%), cassis-flavored liqueur (20%), plant worm-shochu (a Japanese distilled beverage distilled from plant worm; 25%), imo-shochu (a Japanese distilled beverage distilled from sweet potatoes; 25%), vodka (40%), gin (40%), palinka (40%), rum (40%), brandy (40%), and whisky (40%).

In addition, following water/EtOH mixed solutions with different composition were also used:

Water/EtOH volume ratio of 80/20 and 60/40.

Conditions for the sensing experiments are the same with the case under an ambient condition except that the two piezoelectric pumps were switched every 30 seconds.

**B-2 Nanoparticles**

**Figure B-1** is the alcohol content dependence of the parameters extracted from the response signals of the 21 liquid samples measured by the MSS when Aminopropyl-STNPs, Vinyl-STNPs, C18-STNPs, and Phenyl-STNPs were used as a receptor layer material. Here, the parameters were extracted by using Eqs. (1)-(4), and $t_{b}= t_{a}+3$[s], $t_{c}= t_{a}+30$[s], and $t_{d}= t_{a}+33$[s]. Furthermore, in each liquid sample, three peaks where $t_{a}=90, 150,$ and $210$ were used and the 63 data exist in **Fig. B-1**. Except the parameter 2, moderate correlations of parameters with respect to the alcohol content were confirmed for all parameters. **Figure B-2** and **Table B-1** are the training results under a N_2_ environment when Aminopropyl-STNPs, Vinyl-STNPs, C18-STNPs, and Phenyl-STNPs were used as a receptor layer material. The setting is completely the same with the cases under an ambient condition. For the known liquid samples, the prediction by the ML model was successful when a receptor layer material is C18-STNPs or Phenyl-STNPs while Aminopropyl-STNPs and Vinyl-STNPs showed much larger prediction errors as well as the case under an ambient condition.


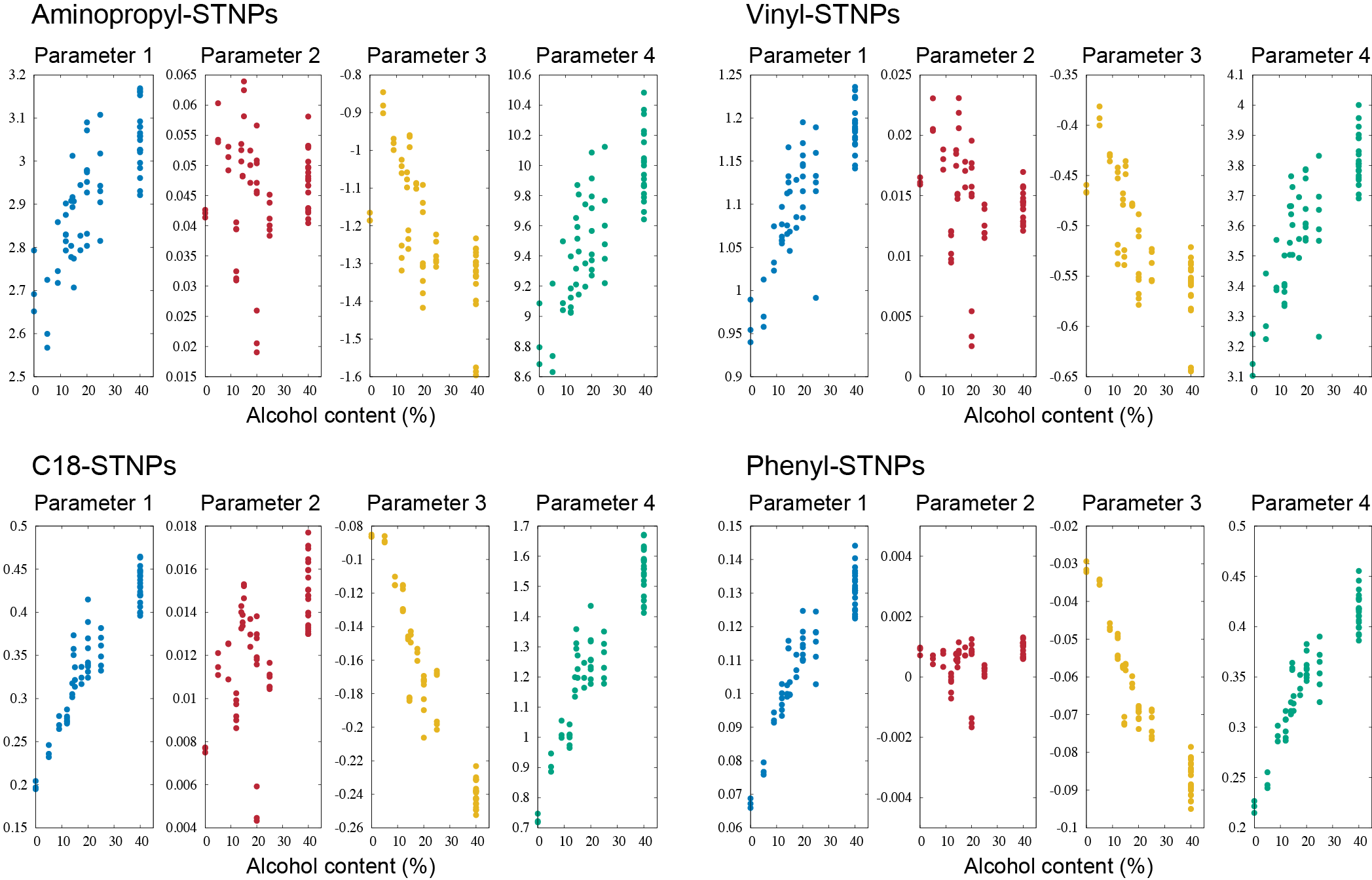


**Fig. B-1** Alcohol content dependence of the parameters extracted from response signals under a N_2_ condition. In each case, the 63 data exist.


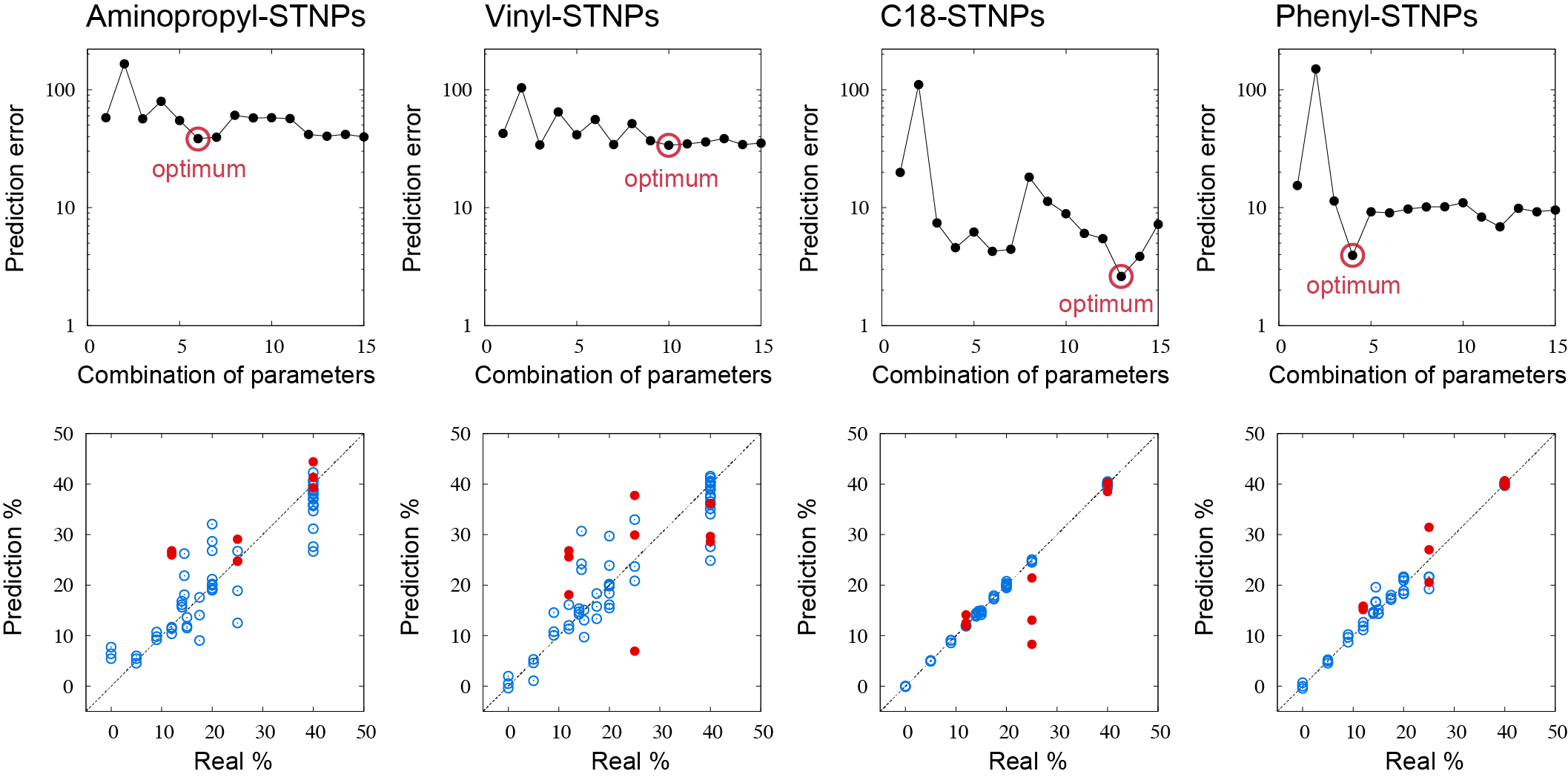


**Fig. B-2** (Top) Prediction errors depending on the combination of four parameters extracted from a response signal under a N_2_ environment. The definition of combinations by the decimal number was explained in caption of **Fig. 8**. (Bottom) Parity plot of predicted alcohol content versus real alcohol content under a N_2_ environment. The blue points represent the known liquid samples which are used to train a ML model. The red points are the unknown liquors: red wine (12%), imo-shochu (25%), and whisky (40%).

**Table B-1** Optimal combination of parameters and optimal prediction error depending on the receptor layer material under a N_2_ environment.

|  | Aminopropyl | Vinyl | C18 | Phenyl |
| --- | --- | --- | --- | --- |
| Parameter 1 |  |  | Use |  |
| Parameter 2 | Use | Use |  |  |
| Parameter 3 | Use |  | Use | Use |
| Parameter 4 |  | Use | Use |  |
| Prediction error | 38.5028 | 33.7003 | 2.6086 | 3.9367 |

**B-3 Polymers**

**Figure B-3** is the alcohol content dependence of the parameters extracted from the response signals of the 21 liquid samples measured by the MSS when Polysulfone and Polycaprolactone were used as a receptor layer material. Moderate correlations of parameters with respect to the alcohol content were confirmed for all parameters. **Figure B-4** and **Table B-2** are the training results under a N_2_ environment when Polysulfone and Polycaprolactone were used as a receptor layer material. The setting is completely the same with the cases under an ambient condition. For the known liquid samples, the prediction by the ML model was successful for both cases.

**
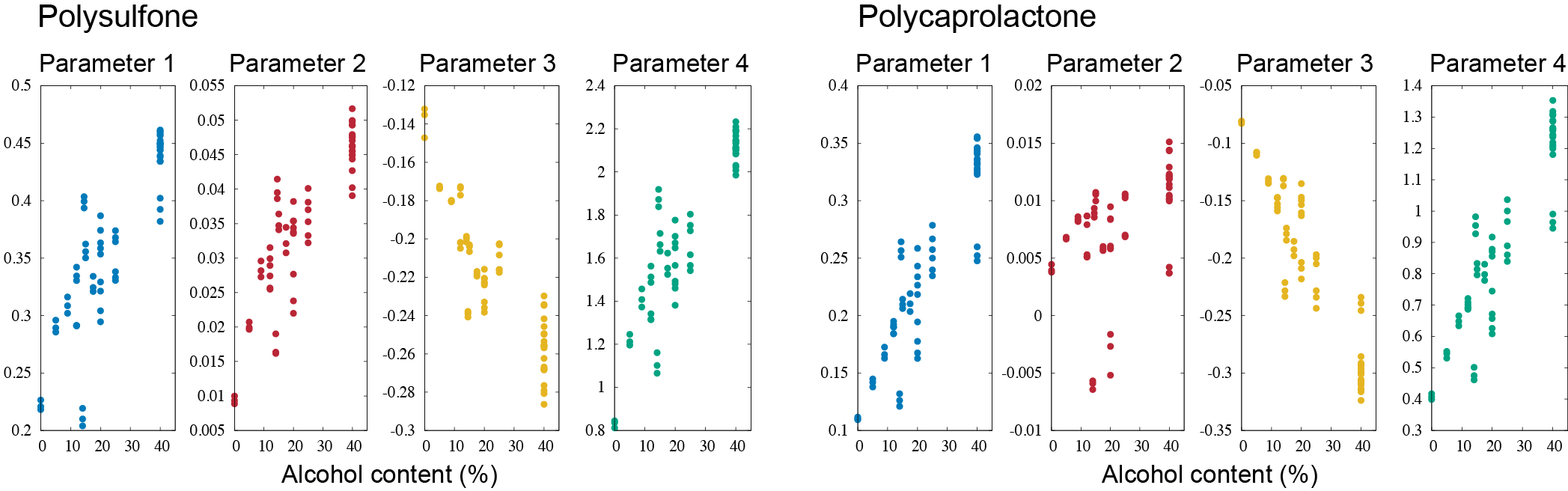
**

**Fig. B-3** Alcohol content dependence of the parameters extracted from response signals under a N_2_ environment. In each case, the 63 data exist.

**
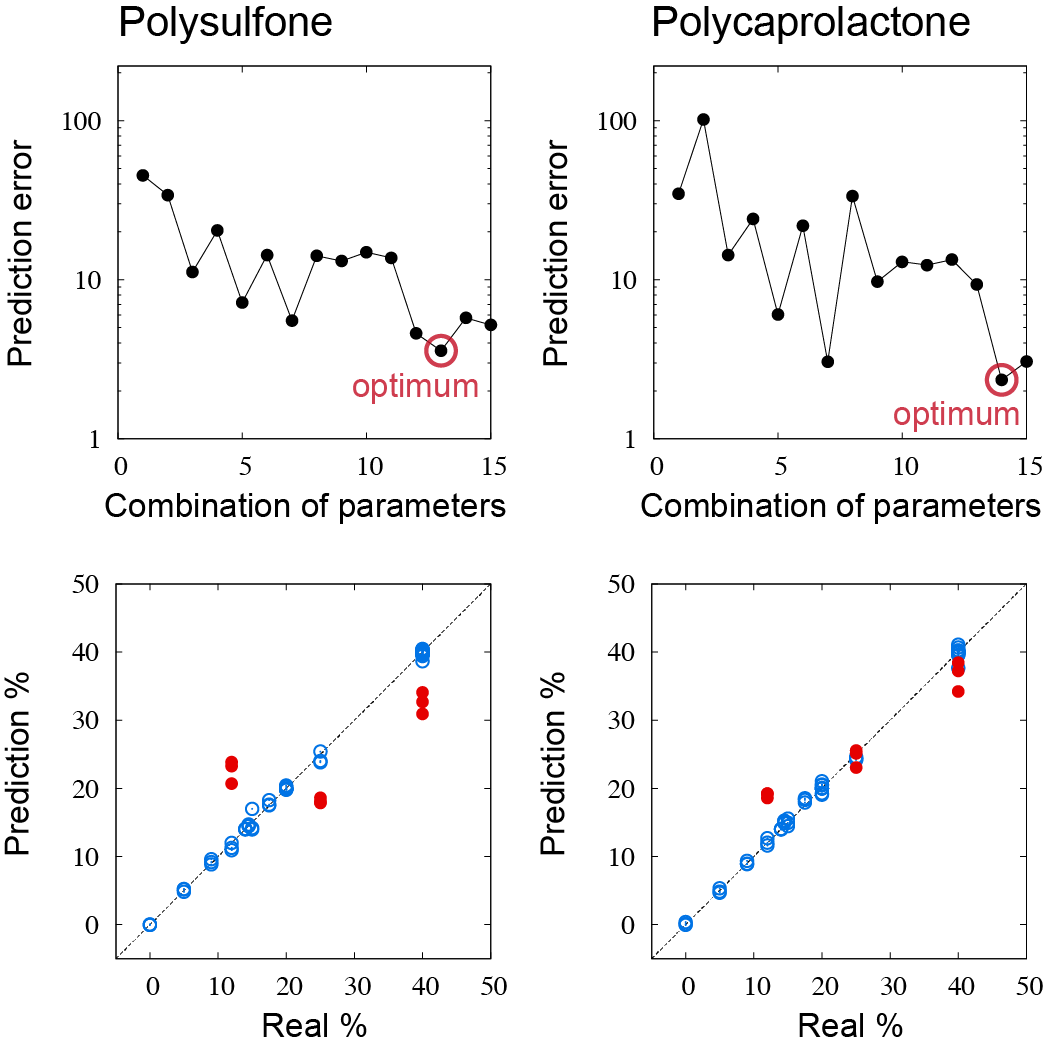
**

**Fig. B-4** (Top) Prediction errors depending on the combination of four parameters extracted from a response signal under a N_2_ environment. The definition of combinations by the decimal number was explained in caption of **Fig. 8**. (Bottom) Parity plot of predicted alcohol content versus real alcohol content under an ambient condition. The blue points represent the known liquid samples which are used to train a ML model. The red points are the unknown liquors: red wine (12%), imo-shochu (25%), and whisky (40%).

**Table B-2** Optimal combination of parameters and optimal prediction error depending on the receptor layer material under a N_2_ environment.

|  | Polysulfone | Polycaprolactone |
| --- | --- | --- |
| Parameter 1 | Use |  |
| Parameter 2 |  | Use |
| Parameter 3 | Use | Use |
| Parameter 4 | Use | Use |
| Prediction error | 3.5752 | 2.3476 |
